# Occurrence, source apportionment, and ecological risk assessment of organophosphate esters in surface sediment from the Ogun and Osun Rivers, Southwest Nigeria

**DOI:** 10.1007/s11356-023-31125-z

**Published:** 2023-11-24

**Authors:** Muideen Remilekun Gbadamosi, Adeyemi Lawrence Ogunneye, David Olaoluwa Jegede, Mohamed Abou-Elwafa Abdallah, Stuart Harrad

**Affiliations:** 1 Faculty of Health and Life Sciences, Coventry University, Coventry, CV1 5FB UK; 2 Department of Chemical Sciences, Tai Solarin University of Education, Ijebu-Ode, Ogun State Nigeria; 3 Chemistry Unit, Department of Basic Science, Babcock University, Ilishan-Remo, Ogun State Nigeria; 4 School of Geography, Earth, and Environmental Sciences, University of Birmingham, Birmingham, B15 2TT UK

**Keywords:** Organophosphate esters, Sediments, Nigeria, Toxicity, Risk quotient

## Abstract

Organophosphate esters (OPEs) are synthetic chemicals widely used as e.g., flame retardants and plasticisers in various consumer products. Due to the toxicity of OPEs in aquatic ecosystems, exposure of fauna and flora to these compounds is of potential concern. In this study, the concentrations, profiles, sources, and ecological risk of eight OPEs were investigated in the sediments from the two major rivers in southwest Nigeria. Concentrations of ∑OPEs in surface sediments were in the range 13.1 – 2110 ng/g dry weight (dw) (median: 378 ng/g dw) in the Ogun River and 24.7—589 ng/g dw (median: 174 ng/g dw) in the Osun River. These concentrations are broadly within the range of those reported in surface sediment in previous studies conducted in other locations around the world. Tris (2-butoxyethyl) phosphate (TBOEP) was the dominant OPE in the sediment samples with a median concentration of 337 and 126 ng/g dw for the Ogun and Osun Rivers respectively, while tri-n-butyl phosphate (TnBP) was not detected in any sample. Excluding TBOEP, the chlorinated organophosphate esters: tris(2-chloroethyl) phosphate (TCEP), tris(2-chloro-propyl) phosphate (TCIPP), and tris(1,3-dichloro-2-propyl) phosphate (TDCIPP) were the dominant OPEs in the Osun River, while the aryl-OPEs: triphenyl phosphate (TPHP), 2-ethylhexyl diphenyl phosphate (EHDPP), and tri-*m*-tolyl phosphate (TMTP) were dominant in the Ogun River. Under a median exposure scenario, moderate ecological risk was predicted from exposure to TCIPP in the Osun River. In contrast, under a high exposure scenario, concentrations of TDCIPP (risk quotient, RQ = 5.33—5.37) constituted a high ecological risk in both rivers, with moderate risks observed for TBOEP (RQ = 0.022—0.18) and TCIPP (RQ = 0.097 – 0.16). Therefore, the risk to aquatic organisms from concomitant exposure to mixtures of OPEs in freshwater ecosystems requires further investigation.

**Graphical Abstract:**

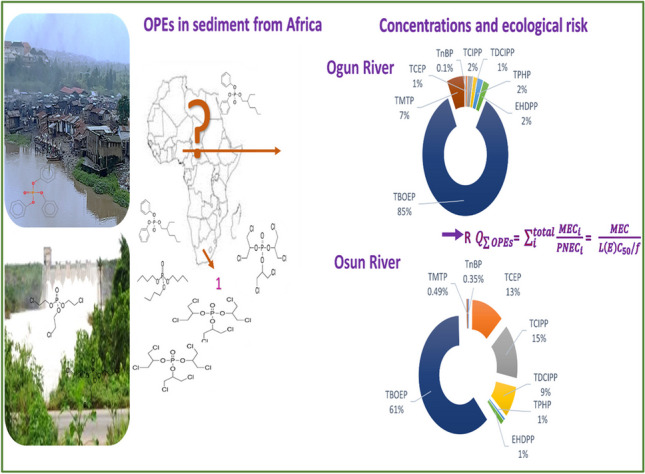

**Supplementary Information:**

The online version contains supplementary material available at 10.1007/s11356-023-31125-z.

## Introduction

Organophosphate esters (OPEs) are additive high production volume chemicals widely used in applications such as plasticisers and flame retardants in a variety of consumer and industrial products, including textiles, plastics, electronics, furniture, varnishes, nail polishes, building materials, and hydraulic fluids (Van der Veen and de Boer 2012; Schmidt et al. 2020; Zhang et al. 2021a; Al-Omran et al. 2021). OPEs are additive chemicals that may be emitted from products into various environmental compartments, leading to wildlife and humans exposure (Xu et al. 2016; Brommer and Harrad 2015; Hou et al. 2016; Gbadamosi et al. 2023a,b). The potential increased application of OPEs in various consumer products because of bans on the production and use of brominated flame retardants (Zhou et al. 2021) has likely contributed to the ubiquitous presence of OPEs in various environmental matrices (Gbadamosi et al. 2021a;^2022^; Brommer and Harrad 2015). Several toxicological studies have reported OPEs to elicit various adverse effects in humans and other animals. For example, tris(2-chloroethyl) phosphate (TCEP), tris (1-chloro-2-propyl) phosphate (TCIPP), and tris(1,3-dichloro-2-propyl) phosphate (TDCIPP) are neurotoxic and carcinogenic (Wei et al. 2015; van den Veen and de Boer 2012), while TCEP, TCIPP, TDCIPP, and triphenyl phosphate (TPHP) have been linked with oestrogen disruption and thyroid hormone (Zhang et al. 2016). Moreover, tributoxyethyl phosphate (TBOEP) has been found to decrease red blood cell cholinesterase activity, while tri-n-butyl phosphate (TnBP), trimethyl phosphate (TMP), and triethyl phosphate (TEP) were found to disrupt thyroid hormones through activation of nuclear receptors (Zhang et al. 2016; Wei et al. 2015; Wang et al. 2014). With respect to epidemiological studies, exposure to OPEs has been associated with altered hormone levels and semen quality (Meeker and Stapleton 2010), adverse reproductive outcomes (Ingle et al. 2018; Carignan et al. 2017), allergic symptoms, adverse reproductive impact, increased oxidative stress (Araki et al. 2018; Ingle et al. 2018; Carignan et al. 2017; Bamai et al. 2019).

Both aryl-OPEs, e.g., 2-ethylhexyl diphenyl phosphate (EHDPP) and alkyl-OPEs e.g., tris(2-ethylhexyl) phosphate (TEHP), are highly hydrophobic, with high log K_OW_ values (> 5), and consequently high bioaccumulation and biomagnification potential in aquatic food webs (Wu et al. 2022; Bekele et al. 2019; Wang et al. 2019). Moreover, the chlorinated OPEs (Cl-OPEs) are highly persistent (half-lives exceeding 1,000 h) and moderately water-soluble chemicals (log K_OW_ < 4), that are discharged into the aquatic environment through activities such as sewage treatment (Wu et al. 2022). Aquatic ecosystems provide important reservoirs for various persistent organic pollutants (Chen et al. 2021; Wang et al. 2021) and specifically, OPEs can exist both in the dissolved phase and sorbed to both suspended and surficial sediment (Zhong et al. 2018). Sediment is considered as a ‘’source’’ and ‘’sink’’ of anthropogenic pollutants an aquatic ecosystem (Liang et al. 2021; Hu et al. 2020; Gbadamosi et al. 2021b). OPEs in coastal environment undergo complex transport processes and accumulated in the various aquatic flora and fauna; alternately, sediment can act as a secondary source of hydrophobic contaminants in aquatic environment (Zeng et al. 2005; Liang et al. 2021). Given these considerations, it is important to understand better the levels and fate of OPEs in aquatic environments.

Recently, there have been several reports of the concentrations of ∑OPEs in river sediment (Liang et al. 2021; Chokwe and Okonkwo 2019; Ji et al. 2022; Yadav et al. 2018; Cristale et al. 2013). The highest reported concentrations of ∑OPEs ranging between 983 – 7450 ng/g and 17 – 4400 ng/g, were obtained in Bagmati River, in Nepal and urban river sediment from Guangzhou, South China (Yadav et al. 2018; Liang et al. 2021). However, despite reports of the presence of OPEs in the aquatic environment in various locations, to date, only one study has documented the presence of OPEs in river sediment from Africa, specifically from the Vaal River catchment in South Africa (∑OPEs range: 67.8 – 278 ng/g dw; median: 120 ng/g dw) (Chokwe and Okonkwo 2019). Given this paucity of data on OPEs in African sediments, the current study was designed to provide a broad and reliable evaluation of the occurrence, fate, and ecological risk of eight OPEs in the sediment of two major rivers (the Ogun and Osun) in southwest Nigeria.

The choice of the Ogun and Osun Rivers for this study is because they represent the major source of water supply and livelihood for fishermen in both Ogun and Osun states in Nigeria. Nevertheless, both rivers received discharges from residential areas. However, the Ogun River was more severely impacted by industrial discharges, urban sewage and waste discharges, pollution from abattoir waste, agricultural activities, and from an e-waste dismantling site nearby. As highlighted above, very scant information is available on the contamination of the aquatic environment with OPEs in Africa, and to the best of our knowledge, this is the first study to report the concentrations, and associated ecological risk of OPEs in riverine sediment in Nigeria and only the second in Africa. Hence, this study aims to: (a) determine the levels and distribution of OPEs in the Ogun and Osun Rivers, (b) examine the potential sources of OPEs in the two rivers and (c) evaluate the ecological risk of the observed OPE contamination to aquatic organisms.

## Materials and methods

### Standards and reagents

Detailed information about the target OPEs are provided in Table S1. Information about the reagents and standards used in this study are provided in “Introduction” section of the supplementary information (SI).

### Study areas and sampling

**Fig. 1:**
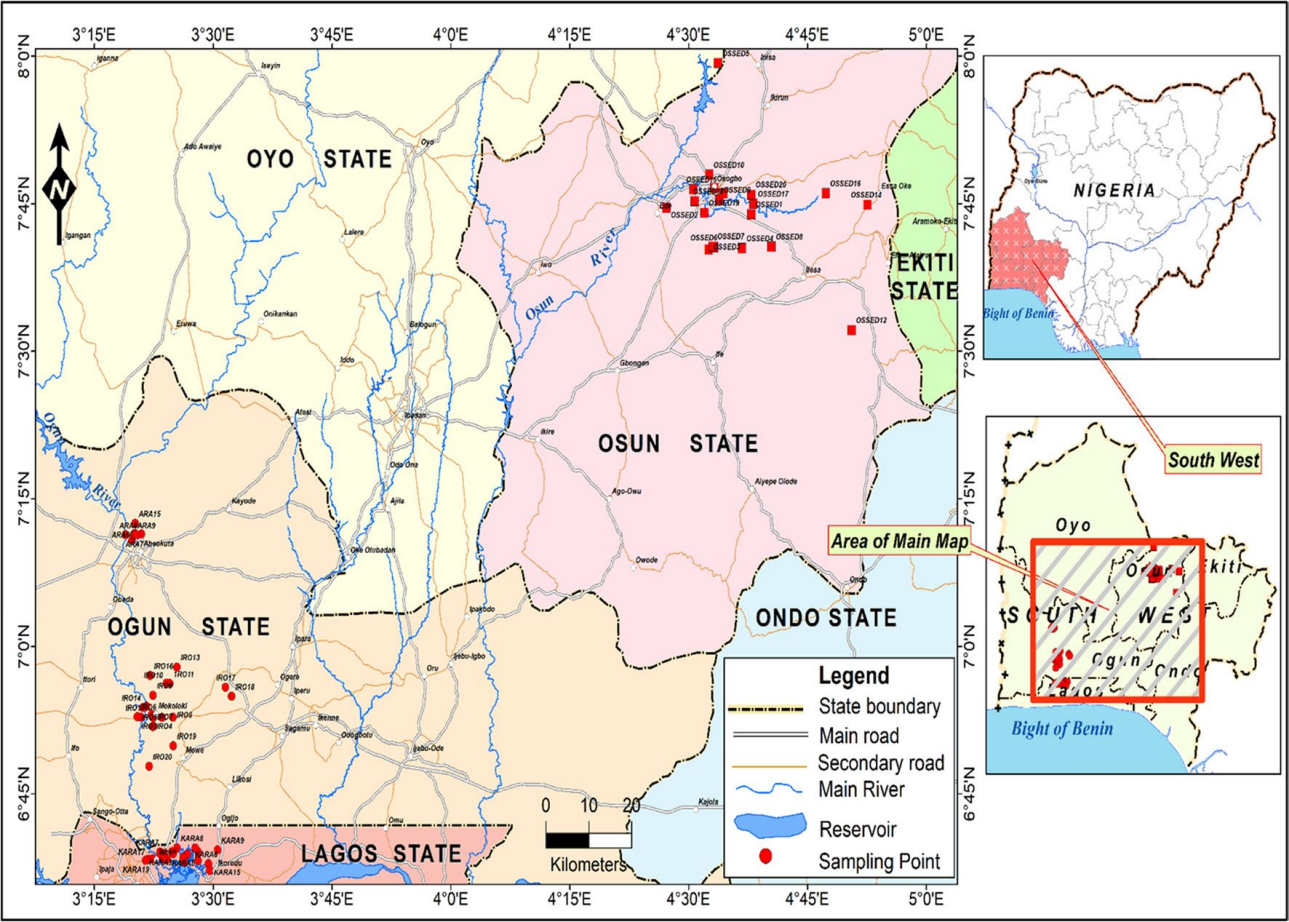
Map of the study area and sampling points

A total of 80 surface sediment samples were collected between September to October 2021. Sixty sediment samples were collected from three locations on the Ogun River as it flows from Abeokuta before emptying to the Lagos Lagoon. These were: Arakanga in Abeokuta, (n = 20), Iro (n = 20), and Kara (n = 20). The remaining 20 sediments were collected from the following locations on the Osun River: Gbodofan/Isale Osun (n = 5), Capital/Halleluyah (n = 5), Aerodrome/Ido Osun (n = 5), and Ede area (n = 5). Sampling locations are indicated on Fig. 1. Our sampling points on the Ogun, especially Kara and Arakanga are highly urbanised and populated, receiving emissions from industrial and/or domestic sources while sampling points on the River Osun have lower population and mainly received domestic discharges.

Sediment samples were collected using a stainless-steel corer pre-washed with dichloromethane before using. After collection, samples were transferred into the aluminium foil and polythene zip lock bag, and stored at -20 °C. The samples were carefully conveyed to the laboratory at the University of Birmingham, where they were freeze dried, ground, and stored in a centrifuge tube prior to extraction and analysis.

### Sample pretreatment and instrumental analysis

Extraction of OPEs from sediment was conducted via ultrasonic assisted extraction (UAE) using a method developed at the University of Birmingham for indoor dust by Brommer et al. (2012) with slight modification. One g of freeze-dried and homogenised sediment was spiked with 50 ng of internal (surrogate) standard mixture (TnBP-d_27_ and TPHP-d_15_) and 1 g of activated copper powder was added to remove elemental sulfur. Samples were extracted in three cycle by sonication with 5 mL of hexane: acetone (1:1, v/v) for 10 min at 40 °C, before centrifugation of the combined extracts at 3500 rpm for 3 min. Following this, extracts were reduced to ~ 1 mL in a clean tube under a gentle stream of high-purity nitrogen. The extract was then passed through a Hypersep Florisil® cartridge conditioned with 2 × 3 mL of hexane, and washed with 10 mL of hexane before elution of target OPEs with 8 mL of ethyl acetate. The eluate was then collected in a clean dry tube, concentrated to incipient dryness under a gentle nitrogen flow and the residue re-dissolved with 100 µL of iso-octane containing 250 pg/µL of PCB-62 as a recovery determination standard (RDS). The final extracts was transferred into a brown vial, stored at -20 °C before injection into GC-EIMS. Retention times and qualification ions are provided as supplementary information (Table S2), with further detailed information on the instrumental analysis described elsewhere (Gbadamosi et al. 2022;Gbadamosi et al. 2023a).

### Quality assurance/quality control (QA/QC)

Appropriate measures were put in place to avoid cross contamination as all used glassware was washed, rinsed with distilled Milli-Q water, baked at 450 °C for 6 h, before rinsing with acetone and dichloromethane before use. A procedural blank was run for each five samples (one batch) and only one compound (TCEP) consistently detected in all procedural blanks at an average concentration of 0.29 ± 0.14 ng/g (Table S3). Where this represented between 5 and 20% of the concentrations detected in samples, blank-correction of concentrations in samples was carried out. The relative response factors (RRFs) for all the target OPEs in the calibration standards (50 – 750 pg/µL) gave the relative standard deviation (RSD) that were < ± 8%. The linear correlation coefficients (r^2^) of the calibration plots were all ˃ 0.99. Average ± standard deviation recoveries of the internal (surrogate) standards in all samples were 80.6 ± 6.0% and 89.5 ± 10.5% for d_15_-TPHP and d_27_-TnBP respectively (Table S3). In addition, recoveries of individual native OPEs were evaluated by spiking sodium sulfate with known amounts of each target OPE and analysing this as a sample. Average ± standard deviation recoveries of all the target OPEs in these spiked sodium sulfate blanks (n = 5) ranged from 82.4 ± 7.2% to 101.1 ± 12.3% (Table S3). The instrumental limit of detection (iLOD) and the instrumental limit of quantification (iLOQ) were calculated based on ten injections of the lowest concentration standards (50 pg/µL), as those concentrations that yielded signal–noise ratios (S/N) of 3:1 and 10:1 respectively (Table S3). For the purposes of statistical evaluation, concentration values where OPEs were not detected in samples, were assigned as the average of the quantification limits (< LOQ = 0.5 × LOQ). OPEs with detection frequencies < 40% were excluded from the statistical analysis.

### Statistical analysis

Descriptive (mean, median, standard deviation, minimum, maximum, and 95^th^ percentile) and multivariate statistics (correlational analysis (CA), principal component analysis (PCA) and hierarchical cluster analysis (HCA) and analysis of variance (ANOVA)) were performed using Microsoft excel 365 and IBM SPSS Statistics 28 (USA) for Windows. PCA was used to apportion and distinguish the sources of OPEs in our samples and conditions such as sampling adequacy and sphericity were satisfy (Table S4). The three principal components (PCs) with eigenvalues ˃ 1 were extracted and retained as the most significant factors. The linear relationships between different variables were investigated using Pearson correlation. Statistical differences were evaluated using t-tests and ANOVA as appropriate, with statistical significance defined as p < 0.05. Cluster analysis was used for grouping of variables with similar characteristics to a new group.

### Ecological risk

Risk quotient (RQ) values were calculated to investigate the individual and combined ecological risk of OPEs using the following equations (Eq. 1 and 2) (Ji et al. 2022; Xing et al. 2018). The RQ is defined as the ratio of the measured environmental concentration (MEC) to the predicted no-effect concentration (PNEC) (Wang et al. 2020a).

1 $$RQ= \frac{MEC}{PNEC}=\frac{MEC}{{L\left(E\right)C}_{50}/f}$$

2 $${RQ}_{\sum OPEs}={\sum }_{i}^{total}\frac{{MEC}_{1}}{{PNEC}_{1}}$$

where *i* represents individual OPEs (for which DF ˃ 40%), f is the assessment factor (European Commission 2003) and L(E)C_50_ refers to as 50% lethal/effective concentration of OPEs which is an expression of the toxicity of OPEs to algae, zebra fish, crustaceans etc. obtained from the literature (Wang et al. 2020a; Yadav et al. 2018). The PNEC values for all target OPEs are those reported elsewhere (Ji et al. 2022; Wang et al. 2020a). The L(E)C_50_ for OPEs were obtained from (Yadav et al. 2018; Verbruggen et al. 2005) and an assessment factor of 10^3^ was used (Yadav et al. 2018; EC 2003). The potential ecological risk was classified as falling into one of the following three levels: low risk (0.01 ≤ RQ ≤ 0.1), moderate/medium risk: (0.1 ≤ RQ ≤ 1.0) and high risk: (RQ ≥ 1.0) respectively (Ji et al. 2022).

## Results and discussion

### Concentrations and profiles of OPEs in surface sediments

**Fig. 2:**
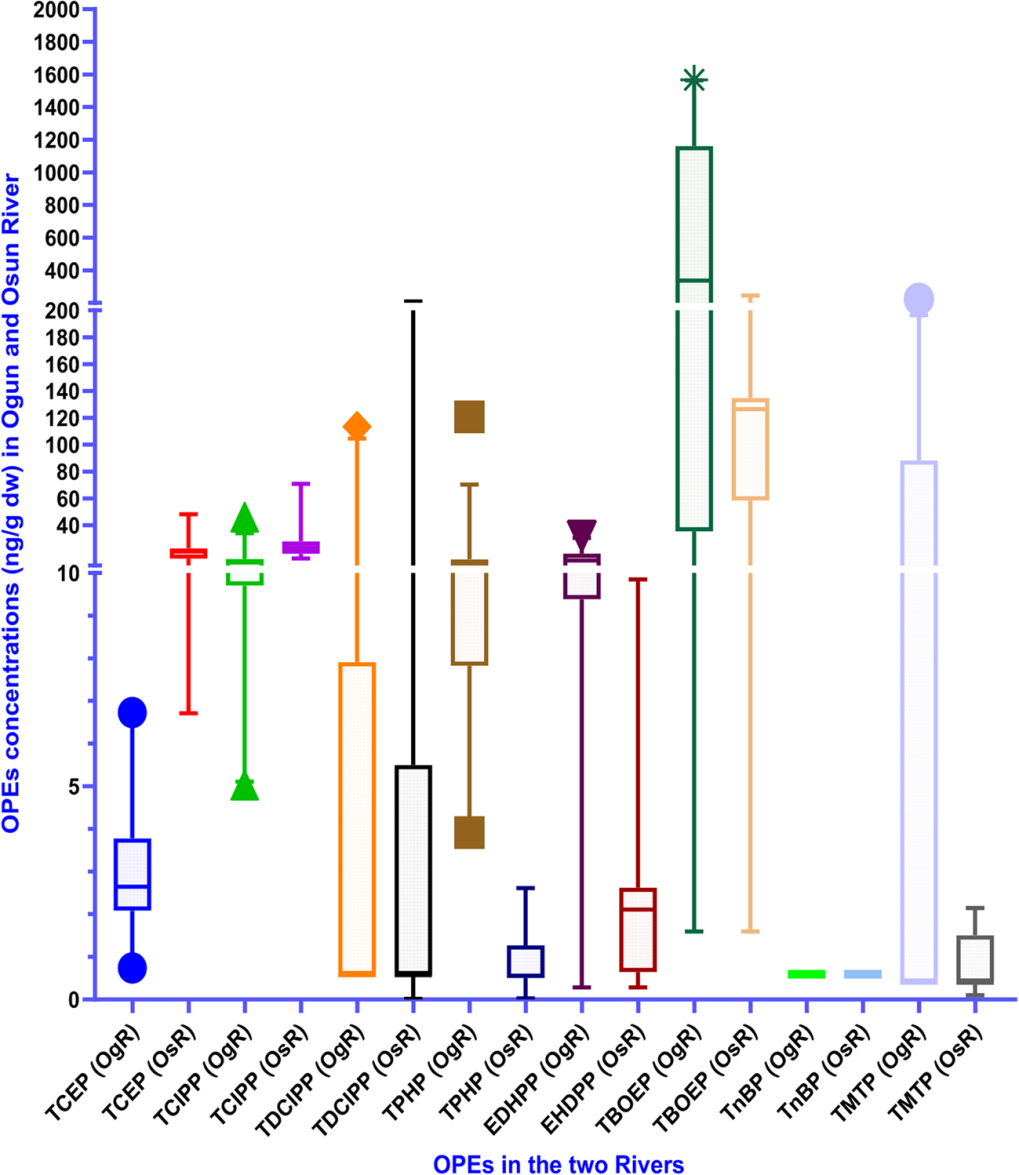
Box plots comparing the concentrations of individual OPE in surface sediment from Ogun River (OgR) and Osun River (OsR). The top and bottom of the central box respectively represent the 25^th^ and 75^th^ percentile concentrations, the middle bold line represents the median, while the top and bottom whiskers respectively represent maximum and minimum concentrations

The summary of the statistics for the concentrations of the OPEs divided into: Cl-OPEs (TCEP, TCIPP, and TDCIPP), aryl-OPEs (EHDPP, TPHP and TMTP) and alkyl-OPEs: TBOEP and TnBP) is presented in Table 1. Detection frequencies varied from 42—100% for Cl-OPEs, 48 – 100% for aryl-OPEs, and for TBOEP from 80 – 92%. TnBP was not detected in any sample. The median and range of concentrations of ∑_8_OPEs in the Ogun River (median: 660; range: 13.1 – 2110 ng/g dw) exceeded those in the Osun River (median: 169; range: 24.7 – 589 ng/g dw) (Table 1; Fig. 2). The high level of urbanization is a likely major source of the higher OPE concentrations in the Ogun River compared to the Osun River. Specifically, samples from the Kara portion of the Ogun River tend to be more polluted than those from other regions due to the fact that this sampling site received effluents from wastewater treatment from both industrial and domestic sources, from the nearby abbatoir and from e-waste dismantling points in Lagos State. These are likely potential sources of elevated OPE input to the Ogun River.

**Table 1:** Statistical summary of OPEs concentrations in surface sediment from Ogun and Osun Rivers

Location	Ogun river sediment (n = 60) (ng/g, dry weight)					Osun river sediment (n = 20) (ng/g, dry weight)					
OPE	DF (%)	Mean	Median	Range	95^th^ Percentile	DF (%)	Mean	Median	Range	95^th^ Percentile	Range of OPE concentrations in sediment from previous studies
TCEP	100	3.10	13.1	0.74–6.73	6.18	100	21.5	20.5	6.71–48.3	33.6	ND – 82.1 ^a,b,c^
TCIPP	100	13.1	12.4	5.03–46.1	21.2	100	25.4	23.4	15.3–70.9	35.5	ND – 892 ^d,e^
TDCIPP	42	9.12	0.57	<LOQ-114	50.5	45	14.7	0.57	<LOQ-209	50.1	ND – 47 ^d,f^
TPHP	100	13.2	10.4	3.91–121	21.3	100	0.95	0.56	0.02–2.61	2.31	ND – 537 ^b,c^
EHDPP	92	14.2	13.8	<LOQ-32.1	28.0	75	2.24	2.11	<LOQ-9.85	3.53	ND – 1100 ^f,d^
TnBP	0	<0.59	<0.59	<0.59	<0.59	0	<0.59	<0.59	<0.59	<0.59	ND – 320 ^b,e^
TBOEP	92	563	337	<LOQ-1567	1443	80	103	126	<LOQ-245	179	ND – 1600 g^,h,b,c^
TMTP	48	43.7	0.40	<LOQ-223	153	55	0.83	0.40	<LOQ-2.14	1.92	-
∑_8_OPEs	-	660	378	13.1–2110	1724	-	169	174	24.7–589	307	-
∑Cl-OPEs	-	25.3	26.1	6.91–167	77.8	-	61.6	44.5	23.2–328	119	-
∑Aryl-OPEs	-	71.1	24.6	5.28–376	202	-	4.02	3.07	1.39–14.6	7.76	-
∑Alkyl-OPEs	-	564	338	3.78–1568	1444	-	104	127	3.78–246	180	=

DF = Detection frequency; LOQ = Limit of quantification

(Ref: ^a^Zhang et al. 2020; ^b^Li et al. 2019; ^c^Ji et al. 2022; ^d^ Liao et al. 2020; ^e^ Yadav et al. 2018; ^f^ Liang et al. 2021; ^g^Peverly et al. 2015; ^h^ Chokwe and Okonkwo 2019)

Generally, median concentrations of aryl-OPEs: TPHP, EHDPP and TMTP in the Ogun River (10.4, 13.8 and 0.40 ng/g dw) exceeded the median concentrations of 0.56, 2.11 and 0.40 ng/g dw obtained in the Osun River. The higher concentrations of aryl-OPEs in the Ogun River may be attributed to the proximity of one of the sampling sites to an e-waste dismantling site in Lagos. This may be as a results of the wide application of aryl OPEs such as plasticizer additives in electronic products, baby products and polyvinyl chloride (PVC) (Lu et al. 2017; van der Veen and de Boer 2012). However, for the Cl-OPEs (TCEP, TCIPP and TDCIPP), the median concentrations of 2.64, 12.4, and 0.57 ng/g dw obtained in the Ogun River were between 2 – 8 times lower than the median concentrations of 20.5, 23.4 and 0.57 ng/g dw obtained from the Osun (Table 1; Fig. 2). The higher concentrations of Cl-OPEs in the Osun River may be related to their wide application in various household products such as textile, furniture and floor covering etc. (Wei et al. 2015) and discharges from domestic waste such as paint (Wei et al. 2015). For the alkyl-OPEs, the highest range and median concentrations was obtained in the Ogun River (range: <LOQ – 1567; median: 337 ng/g dw) than in the Osun (range: <LOQ – 245; median: 126 ng/g dw) (Table 1; Fig. 2). TBOEP (median: 337 ng/g dw) was the most abundant OPE measured in the Ogun River, followed by EHDPP (median: 13.8 ng/g dw), TCIPP (median: 12.4 ng/g dw), TPHP (10.4 ng/g dw) and TCEP (median: 2.64 ng/g dw). Meanwhile, in the Osun River, the relative order is: TBOEP (126 ng/g dw) followed by TCIPP (23.4 ng/g dw), TCEP (20.5 ng/g dw), EHDPP (2.11 ng/g dw) and TPHP (0.56 ng/g dw) respectively (Table 1). A paired t-test revealed a statitistical significant difference between the mean concentrations of ∑_8_OPEs in Ogun river (mean: 660 ng/g dw) and Osun River (mean: 169 ng/g dw) sediments (p < 0.05) (Table 1; Fig. 2). Higher OPE concentrations in the Ogun River may arise from both domestic and industrial discharges as well as releases from e-waste dismantling sites. Moreover, t-test comparison revealed statistically significant differences (p < 0.05) in the concentrations of all individual OPEs, with the exception of TDCIPP and TnBP.

### Global comparison of OPEs concentrations in sediments

**Fig. 3:**
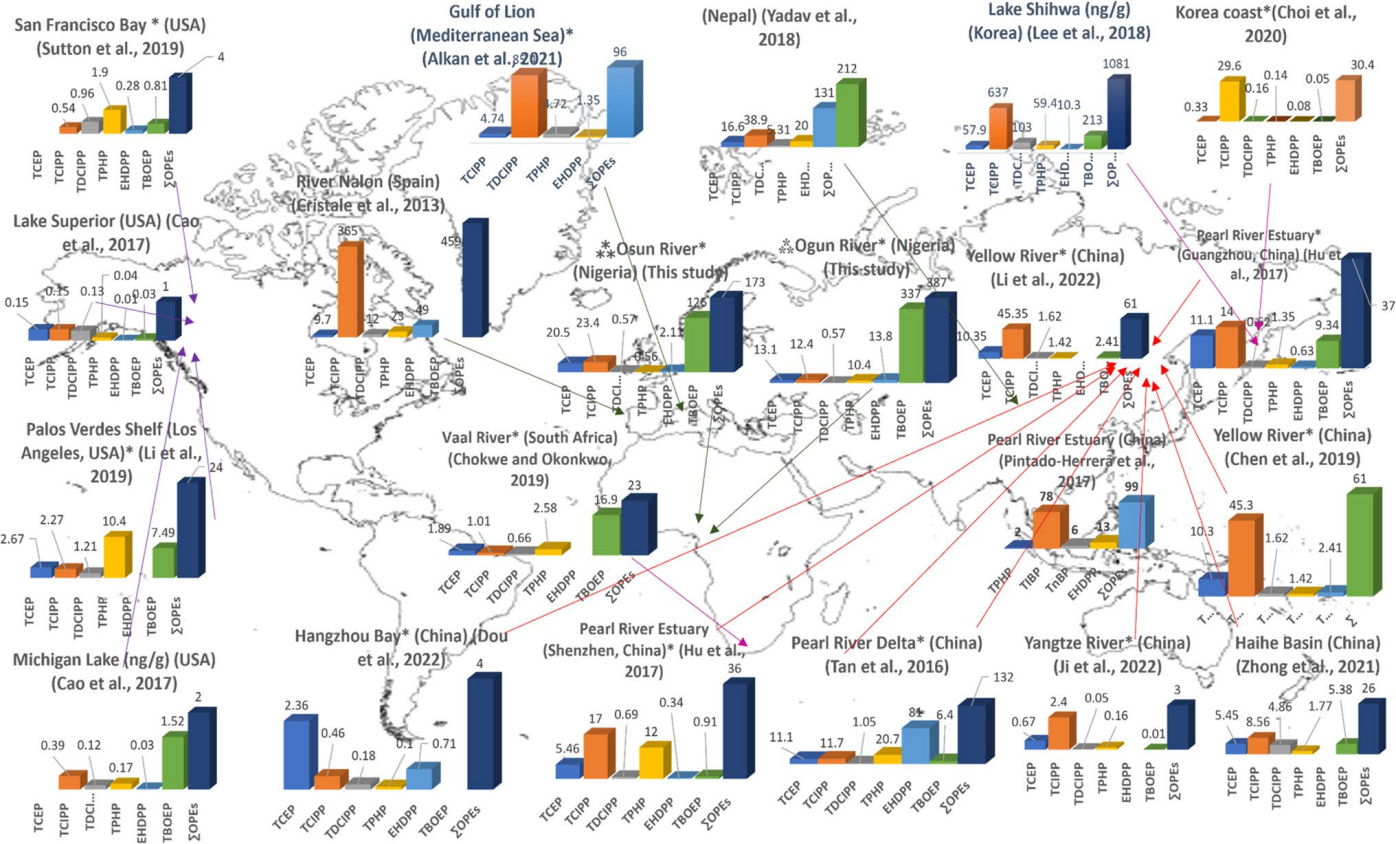
Global comparison of concentrations of OPEs (ng/g dw) in river sediment [NB: * Concentrations reported in median. ⁂ Present study]

Concentrations of ∑_8_OPEs in the sediments from the Ogun River (median: 378 ng/g dw; range: 13.1 – 2110 ng/g dw) and the Osun River (median: 174 ng/g dw; range: 24.7 – 587 ng/g dw) were compared with those reported in studies conducted elsewhere in the world (Table 1 and Table S5). This comparison revealed the concentrations detected in the two rivers in this study fall within the overall range reported for all other studies (Pintado-Herrera et al. 2017; Yadav et al. 2018; Cristale et al. 2013; Liu et al. 2016; Hu et al. 2017; Sutton et al. 2019; Sibiya et al. 2019; Dou et al. 2022; Ji et al. 2022; Choi et al. 2020; Chen et al. 2019; Chokwe and Okonkwo 2019; Peverly et al. 2015; Lee et al. 2018; Alkan et al. 2021) (Table S5; Fig. 3).

There was a marked difference in the OPE patten between the Ogun and Osun Rivers. In the Osun River, Cl-OPEs (median, TCEP: 20.5 ng/g dw; TCIPP: 23.4 ng/g dw; and TDCIPP: 0.57 ng/g dw) were the most abundant OPEs after TBOEP (126 ng/g dw). By comparison, in the Ogun River, aryl-OPEs (TPHP: 10.4 ng/g dw; EHDPP: 13.8 ng/g dw were dominant after TBOEP (337 ng/g dw). The median concentrations of the Cl-OPEs: TCEP (2.64 and 20.5 ng/g dw), TCIPP (12.4 and 23.4 ng/g dw), and TDCIPP (0.57 ng/g dw) in the Ogun and Osun Rivers were comparable to, but tending towards the lower range of values reported in studies elsewhere (Yadav et al. 2018; Liu et al. 2016, Lee et al. 2018; Sibiya et al. 2019; Peverly et al. 2015; Ji et al. 2022; Luo et al. 2020; Mo et al. 2019) (Table S5; Fig. 3). These differences in OPE distribution pattern are likely a result of the different sources impacting the Ogun River compared to the Osun River. The concentrations of TCIPP obtained in this present study were about two order of magnitide lower than those reported in river sediments from Norway (63 – 16,000 ng/g) (Green et al. 2008). The median concentrations of aryl-OPEs: TPHP, EHDPP and TMTP from Ogun River (10.4, 13.8 and 0.40 ng/g dw) exceeded those in the Osun River (0.56, 2.11 and 0.40 ng/g dw). These were comparable to those reported in sediment from Lake Gerencuo (You et al. 2022), below the value reported from sediment in Kathmandu Valley (median TPHP: 20.0 and EHDPP: 131 ng/g dw) (Yadav et al. 2018), Shihwa creeks (TPHP: 59.4 ± 83.0; EHDPP: 10.3 ± 14.8) (Lee et al. 2018), as well as in the Chicago sanitary and ship canal (TPHP: 18 – 170; EHDPP: 28 – 690 ng/g dw) (Peverly et al. 2015) (Table S5; Fig. 3). Concentrations of the aryl-OPEs obtained in Osun River sediment samples exceeded those reported in previous studies (Liao et al. 2020; Ji et al. 2022; Fang et al. 2022; Luo et al. 2020; Giulivo et al. 2017; Zhang et al. 2021a; Wang et al. 2020a). For the alkyl-OPEs, TBOEP was the dominant OPE in the two rivers, while TnBP was not detected in any sample in this study. The median and range of TBOEP concentrations in the Ogun River (median: 337; range: <LOQ – 1567 ng/g dw) were higher than in the Osun River (median: 126; <LOQ – 245 ng/g dw) (Table 1). The median and range of concentrations fall within the range of values reported in previous studies (Peverly et al. 2015; Xing et al. 2018; Ji et al. 2022; Luo et al. 2020; Liao et al. 2020) (Table S5; Fig. 3).

### Potential sources of OPEs in the two rivers

**Fig. 4:**
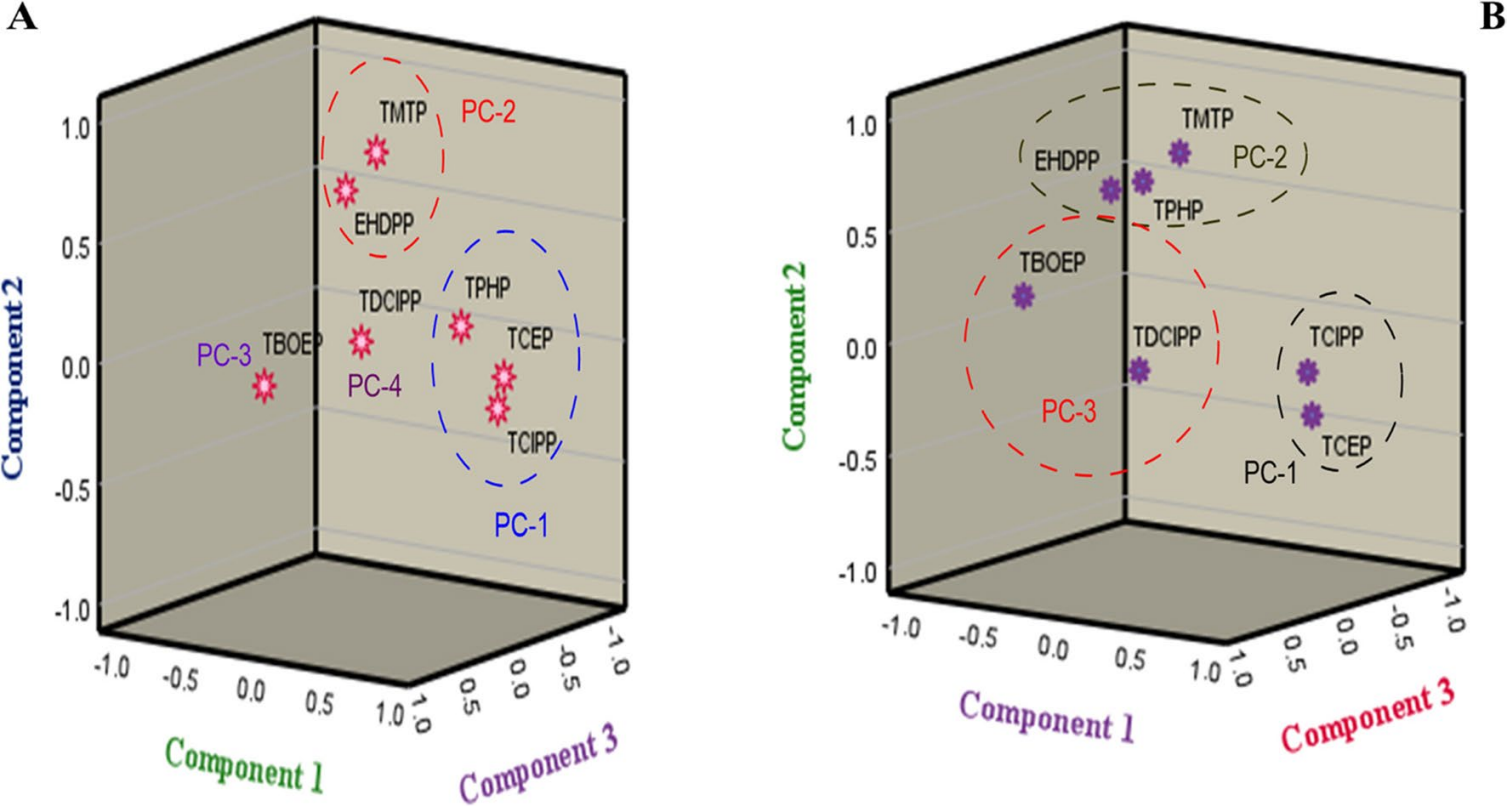
Graphical illustrations of principal components for (**a**) the Ogun River and (**b**) the Osun River

Concentrations of individual OPEs in samples were used to try and identify OPEs with common sources in our sediment samples (Table S8a and S8b). The correlation results from Ogun River sediment showed a significant positive correlation between TCEP and both TCIPP and TPHP (r = 0.850 and 0.696; p < 0.01) (Table S8a). A significant positive correlation was also obtained between TCIPP and TPHP (r = 0.581; p < 0.01) (Table S78a). By comparison, for Osun River sediment, only TCEP and TCIPP show a significant positive correlation with each other (r = 0.790; p < 0.01) (Table S8b). Such positive correlations between these OPEs suggest a common contamination source or sources and/or similar environmental fate and behaviour of these compounds. These findings were supported by the results of a principal component analysis (PCA) (Fig. 4a-b). For the Ogun River, the initial dimension of all the data set produces four components which account for 82% of the total variation (Table S9a; Fig. 4a) and three components which explained 78% of the total variation for Osun River sediment respectively (Table S9b; Fig. 4b). The loadings, eigenvalues, variance, and the cumulative variance of all the extracted components are listed in Table S9a-b. For the Ogun River, the first principal component (PC-1) accounted for 34.7% of the total variation and was driven mainly by TCEP (0.950), TCIPP (0.909) and TPHP (0.829) (Table S9a). This corroborates the results from the correlation analysis where a positive significant correlation existed between these compounds and the same trends was obtained for the Osun River, where PC-1 explained 30.9% of the whole variation with high loading value on TCIPP (0.926) and TCEP (0.887).

TCEP, TCIPP and TPHP are widely used in the production of polyurethane foam, plastics for cables and other construction materials (Boor et al. 2010; van der Veen and de Boer 2012; You et al. 2022), as well as anticorrosion coatings for wood (Ingerowski et al. 2001). This indicates factor 1 arises from mixed pollution sources such as industrial discharges and atmospheric deposition, emissions from the e-waste dismanting site in the Ogun River and from domestic sources in the Osun River (Han et al. 2022; Meng et al. 2020; Wang et al. 2020b; You et al. 2022; Zhang et al. 2021b).

The second principal component (PC-2) from both the Ogun and Osun Rivers respectively explained 17.4 and 26.7% of the total variation of the dataset and was mainly loaded heavily mainly on the aryl-OPEs: Ogun River: TMTP (0.792) and EHDPP (0.721); Osun River: TMTP (0.838), TPHP (0.642) and EHDPP (0.640) (Fig. 4a-b; Table S9a-b). This factor suggests that Ogun and Osun river receives OPE inputs from emission from electronics and textile production companies in this areas (Zhang et al. 2022; van der Veen and de Boer 2012). The third component (PC-3) accounted for 15.1 and 20.3% of the total variance in the data for the Ogun and Osun River sediments respectively and was loaded significantly on TBOEP (0.937) for the Ogun River, and TDCIPP (0.842) and TBOEP (0.762) for the Osun River sediment (Fig. 4a-b; Table S9a-b). TBOEP is commonly used as a plasticizer in polyvinyl chloride (PVC) and as floor finish (Brandsma et al. 2014; Gbadamosi et al. 2023b). Therefore this factor may indicate diffuse urban inputs from the built environment in which PVC and floor finishes are widely used. The last PC-4 accounts for 14.7% of the total variance and heavily loaded on TDCIPP (0.97) for the Ogun River (Table S9a, Fig. 4a), suggesting a distinct source of TDCIPP to the Ogun River.

We also examined the similarity between sediment contamination with the target OPEs using cluster analysis. The results obtained from the cluster analysis (Fig. S1a-b) shows three distinctive clusters with varied minor-major similarities between the OPEs for both Rivers. In the Ogun River, the first cluster (Cluster-I) consisted of TCIPP and TPHP with major similarity with EHDPP and slightly higher similarity with TCEP (Fig. S1a). Cluster-II consisted of TDCIPP with major differences to TBOEP and TMTP in Cluster-III. The same trends were obtained for the Osun River, where clusters I and II consisted of the Cl-OPEs: (TCEP, TCIPP, and TDCIPP) and aryl-OPEs: (TPHP, EHDPP, and TMTP) with major differences from the alkyl-OPE TBOEP. This showed that the Cl-OPEs and the aryl-OPEs considered in this study shared mixed and overlapping contamination sources in the sediment samples and that only TBOEP has a distinct contamination source, likely to its use in floor finishing products or as PVC.

### Potential ecological risk assessment

We evaluated the potential ecological risks posed by our target OPEs in sediment from the Ogun and Osun rivers based on risk quotient (RQ) values calculated under median and high-end concentration scenarios (95^th^ percentile) (Table 2; Table S10). Under the median concentration scenario, RQ values showed no risk for aquatic organisms for TCEP (4.3 × 10^–4^ and 3.3 × 10^–3^), low risk for TPHP (1.9 × 10^–2^ and 1.0 × 10^–3^), EHDPP (2.7 × 10^–2^ and 4.2 × 10^–3^), and TBOEP (4.2 × 10^–2^ and 1.6 × 10^–2^) in Ogun and Osun River (Table 2). Interestingly, the Osun River has a moderate risk for TCIPP (0.11) whereas the Ogun River has a low risk for TCIPP (5.7 × 10^–2^) (Table 2; Table S10). The calculated values of RQ under the high concentration scenario, revealed a high risk for TDCIPP (5.37 and 5.33) in both rivers, as well as a moderate risk for TBOEP (0.18) in the Ogun River and for TCIPP (0.16) in the Osun River (Table 2; Table S10). This showed that the ecological risk to aquatic organisms to OPEs in the sediment from the two rivers varies from low to moderate risk.

**Table 2:** Risk quotient values of OPEs in surface sediments from the Ogun and Osun rivers

Location	Concentration Scenario	TCEP	TCIPP	TDCIPP	TPHP	EHDPP	TBOEP	TMTP^a^
Ogun river sediments (n = 60)	Median	4.3 × 10^–4^	5.7 × 10^–2^	6.1 × 10^–2^	1.9 × 10^–2^	2.7 × 10^–2^	4.2 × 10^–2^	-
	High	1.0 × 10^–3^	9.7 × 10^–2^	**5.37**	3.9 × 10^–2^	5.6 × 10^–2^	**0.18**	-
Osun river sediments (n = 20)	Median	3.3 × 10^–3^	**0.11**	6.1 × 10^–2^	1.0 × 10^–3^	4.2 × 10^–3^	1.6 × 10^–2^	-
	High	5.5 × 10^–3^	**0.16**	**5.33**	4.3 × 10^–3^	7.0 × 10^–3^	2.2 × 10^–2^	-

^a^ RQ values for TMTP not calculated as concentrations in all samples < LOQ

High risk (RQ > 1) and moderate risk (0.1 > RQ < 1) instances in red and yellow respectively

## Conclusion

In this study, the concentrations, relative abundance, and potential ecological risk of OPEs in surface sediments from two major rivers in southwest Nigeria were investigated. Seven target OPEs were frequently detected in the sediments from both rivers with concentrations of ∑_8_OPEs ranging from 13.1 to 2110 ng/g dw (median: 378 ng/g dw) in the Ogun River and between 24.7 and 589 ng/g dw (median: 174 ng/g dw) in the Osun River (including TnBP that was not detected in any of the samples). These concentrations were comparable to and at the lower end of the range of those reported in previous studies elsewhere. Median concentrations of ∑OPEs detected in the Ogun River were about twice those in the Osun. However, the OPE profile varied between the two rivers. Specifically, the concentrations of TCEP, TCIPP, and TDCIPP were higher in the Osun River; while concentrations of TPHP, EHDPP, and TMTP were higher in the Ogun River. For TBOEP, concentrations in the Ogun River exceeded those in the Osun. An ecological risk assessment showed that the 95^th^ percentile concentrations of TDCIPP posed high risk to the aquatic organisms in the two rivers, while both median and 95^th^ percentile concentrations of TCIPP and the 95^th^ percentile concentration of TBOEP posed moderate ecological risk. Continued monitoring of the presence of OPEs in these and similar waterways is recommended, given continued reports of the potential adverse environmental impacts of such contaminants.

### Supplementary Information

Below is the link to the electronic supplementary material.

Supplementary file1 (DOCX 401 KB)

## Data Availability

All data generated or analyzed during this study are included in this published article.

## References

[CR1] Alkan N, Alkan A, Castro-Jiménez J, Royer F, Papillon L, Ourgaud M, Sempéré R (2021). Environmental occurrence of phthalate and organophosphate esters in sediments across the Gulf of Lion (NW Mediterranean Sea). Sci Total Environ.

[CR2] Al-Omran LS, Gbadamosi MR, Stubbings WA, Drage DS, Abdallah MA, Harrad S (2021). Organophosphate esters in indoor and outdoor dust from Iraq: implications for human exposure. Emerg Contam.

[CR3] Araki A, Bastiaensen M, Bamai YA, Van den Eede N, Kawai T, Tsuboi T, Ketema RM, Covaci A, Kishi R (2018). Associations between allergic symptoms and phosphate flame retardants in dust and their urinary metabolites among school children. Environ Int.

[CR4] Bamai AY, Bastiaensen M, Araki A, Goudarzi H, Konno S, Ito S, Miyashita C, Yao Y, Covaci A, Kishi R (2019). Multiple exposures to organophosphate flame retardants alter urinary oxidative stress biomarkers among children: the Hokkaido study. Environ Int.

[CR5] Bekele TG, Zhao H, Wang Q, Chen J (2019). Bioaccumulation and trophic transfer of emerging organophosphate flame retardants in the marine food webs of Laizhou Bay, North China. Environ Sci Technol.

[CR6] Boor SL, Nobuko Y, Minato E, Masayuki M (2010) A mixture of triethylphosphate and ethylene carbonate as a safe additive for ionic liquid-based electrolytes of lithiumion batteries. J Power Sources 195(21). 10.1016/j.jpowsour.2010.05.040

[CR7] Brandsma SH, de Boer J, van Velzen MJM, Leonards PEG (2014). Organophosphorus flame retardants (PFRs) and plasticizers in house and car dust and the influence of electronic equipment. Chemosphere.

[CR8] Brommer S, Harrad S (2015). Sources and human exposure implications of concentrations of organophosphate flame retardants in dust from UK cars, classrooms, living rooms, and offices. Environ Int.

[CR9] Brommer S, Harrad S, Eede NVD, Covaci A (2012). Concentrations of organophosphate esters and brominated flame retardants in German indoor dust samples. J Environ Monit.

[CR10] Carignan CC, Mínguez-Alarcón L, Butt CM, Williams PL, Meeker JD, Stapleton HM, Toth TL, Ford JB, Hauser R, EARTH Study Team (2017). Urinary Concentrations of Organophosphate Flame Retardant Metabolites and Pregnancy Outcomes among Women Undergoing *in Vitro* Fertilization. Environ Health Perspect.

[CR11] Chen MQ, Gan ZW, Qu B, Chen SB, Dai YY, Bao XM (2019). Temporal and seasonal variation and ecological risk evaluation of flame retardants in seawater and sediments from Bohai Bay near Tianjin, China during 2014 to 2017. Mar Pollut Bull.

[CR12] Chen ZK, An C, Chen X, Taylor E, Bagchi A, Tian X (2021). Inexact inventory-theory-based optimization of oily waste management system in shoreline spill response. Sci Total Environ.

[CR13] Choi W, Lee S, Lee HK, Moon HB (2020). Organophosphate flame retardants and plasticizers in sediment and bivalves along the Korean coast: occurrence, geographical distribution, and a potential for bioaccumulation. Mar Pollut Bull.

[CR14] Chokwe TB, Okonkwo JO (2019). Occurrence, distribution and ecological risk assessment of organophosphorus flame retardants and plasticizers in sediment samples along the Vaal River catchment, South Africa. Emerg Contam.

[CR15] Cristale J, Katsoyiannis A, Sweetman AJ, Jones KC, Lacorte S (2013). Occurrence and risk assessment of organophosphorus and brominated flame retardants in the River Aire (UK). Environ Pollut.

[CR16] Dou W, Zhang Z, Huang W, Wang X, Zhang R, Wu Y, Sun A, Shi X, Chen J (2022). Contaminant occurrence, spatiotemporal variation, and ecological risk of organophosphorus flame retardants (OPFRs) in Hangzhou Bay and east China sea ecosystem. Chemosphere.

[CR17] European Commission (2003) Technical Guidance Document on Risk Assessment in Support of Commission Directive 93/67/EEC on Risk Assessment for New Notified Substances, Commission Regulation (EC) No 1488/94 on Risk Assessment for Existing Substances, Directive 98/8/EC of the European Parliament and of the Council Concerning the Placing of Biocidal Products on the Market. Part II. Accessed 20 June 2022

[CR18] Fang L, Liu A, Zheng M, Wang L, Hua Y, Pan X, Xu H, Chen X, Lin Y (2022). Occurrence and distribution of organophosphate flame retardants in seawater and sediment from coastal areas of the East China and Yellow Seas. Environ Pollut.

[CR19] Gbadamosi MR, Abdallah MAE, Harrad S (2021). A critical review of human exposure to organophosphate esters with a focus on dietary intake. Sci Total Environ.

[CR20] Gbadamosi MR, Abayomi AA, Afolabi TA, Adegboye MA, Bakare HO, Banjoko OO, Ogunneye AL, Ugbomeh IL, Jegede DO, Ajetunmobi AE, Bakare TE (2021). Pollution sources identification, health, and radiological risk assessment of naturally occurring radioisotopes and heavy metals in waste dumpsites in Ijebu-Ode, Ogun State, Southwest Nigeria. Environ Forensics.

[CR21] Gbadamosi MR, Abdallah MAE, Harrad S (2022). Organophosphate esters in UK diet; exposure and risk assessment. Sci Total Environ.

[CR22] Gbadamosi MR, Al-Omran LS, Abdallah MAE, Harrad S (2023). Concentrations of organophosphate esters in drinking water from the United Kingdom: implications for human exposure. Emerg Contam.

[CR23] Gbadamosi MR, Ogunneye AL, Al-Omran LS, Abdallah MA, Harrad S (2023). Presence, source attribution, and human exposure to organophosphate esters in indoor dust from various microenvironments in Nigeria. Emerg Contam.

[CR24] Gbadamosi MR, Moberuagba KH, Abdallah MAE, Harrad S (2023c) Occurrence and dietary exposure to organophosphorus esters in foodstuffs of Nigerian origin. 152:109880

[CR25] Giulivo M, Capri E, Kalogianni E, Milacic R, Majone B, Ferrari F, Eljarrat E, Barceló D (2017). Occurrence of halogenated and organophosphate flame retardants in sediment and fish samples from three European river basins. Sci Total Environ.

[CR26] Green N, Schlabach M, Bakke T, Brevik EM, Dye C, Herzke D, Huber S, Plosz B, Remberger M, Schoyen M, Uggerud HT, Vogelsang C (2008) Screening of selected metals and new organic contaminants 2007. Norwegian Pollution Control Agency

[CR27] Han B, Chen L, Li Y, Yu L, Zhang J, Tao S, Liu W (2022). Spatial distribution and risk assessment of 11 organophosphate flame retardants in soils from different regions of agricultural farmlands in mainland China. Sci Total Environ.

[CR28] Hou R, Xu Y, Wang Z (2016). Review of OPFRs in animals and humans: absorption, bioaccumulation, metabolism, and internal exposure research. Chemosphere.

[CR29] Hu YX, Sun YX, Li X, Xu WH, Zhang Y, Luo XJ, Dai SH, Xu XR, Mai BX (2017). Organophosphorus flame retardants in mangrove sediments from the Pearl River Estuary, South China. Chemosphere.

[CR30] Hu XY, Yang T, Liu C, Jin J, Gao BL, Wang XJ, Qi M, Wei BK, Zhan YY, Chen T, Wang HT, Liu YT, Bai DR, Rao Z, Zhan N (2020). Distribution of aromatic amines, phenols, chlorobenzenes, and naphthalenes in the surface sediment of the Dianchi Lake, China. Front Environ Sci Eng.

[CR31] Ingerowski G, Friedle A, Thummulla J (2001) Chlorinated ethyl and isopropyl phosphoric acid triesters in the indoor environment – an interlaboratory exposure study. Indoor Air 11(433):145–149 10.1034/j.1600-0668.2001.011003145.x 11521497

[CR32] Ingle ME, Minguez-Alarcon L, Carignan CC, Butt CM, Stapleton HM, Williams PL, Ford JB, Hauser R, Meeker JD (2018). The association between urinary concentrations of phosphorous-containing flame retardant metabolites and semen parameters among men from a fertility clinic. Int J Hyg Environ Health.

[CR33] Ji B, Liu Y, Wu Y, Liang Y, Gao S, Zeng X, Yao P, Yu Z (2022). Organophosphate esters and synthetic musks in the sediments of the Yangtze River Estuary and adjacent East China Sea: Occurrence, distribution, and potential ecological risks. Mar Pollut Bull.

[CR34] Lee S, Cho HJ, Choi W, Moon HB (2018). Organophosphate flame retardants (OPFRs) in water and sediment: Occurrence, distribution, and hotspots of contamination of Lake Shihwa, Korea. Mar Pollut Bull.

[CR35] Li J, Wang J, Taylor AR, Cryder Z, Schlenk D, Gan J (2019). Inference of organophosphate ester emission history from marine sediment cores impacted by wastewater effluents. Environ Sci Technol.

[CR36] Liang C, Peng B, Wei G-L, Gong Y, Liu G, Zeng L, Liu L-Y, Zeng EY (2021). Organophosphate diesters in urban River sediment from South China: call for more research on their occurrence and fate in field environment. ACS EST Water.

[CR37] Liao C, Kim U-J, Kannan K (2020). Occurrence and distribution of organophosphate esters in sediment from northern Chinese coastal waters. Sci Total Environ.

[CR38] Liu J, He LX, Zeng XY, Yu ZQ, Ran Y, Sheng GY, Fu JM (2016). Occurrence and distribution of organophosphorus flame retardants/plasticizer in surface sediments from the Pearl River and Dongjiang River. Asian J Ecotox.

[CR39] Lu SY, Li YX, Zhang T, Cai D, Ruan JJ, Huang MZ, Wang L, Zhang JQ, Qiu RL (2017). Effect of E-waste Recycling on Urinary Metabolites of Organophosphate Flame Retardants and Plasticizers and Their Association with Oxidative Stress. Environ Sci Technol.

[CR40] Luo Q, Gu LY, Wu ZP, Shan Y, Wang H, Sun LN (2020). Distribution, source apportionment and ecological risks of organophosphate esters in surface sediments from the Liao River, Northeast China. Chemosphere.

[CR41] Meeker JD, Stapleton HM (2010). House dust concentrations of organophosphate flame retardants in relation to hormone levels and semen quality parameters. Environ Health Perspect.

[CR42] Meng WK, Li JH, Shen JY, Deng YR, Letcher RJ, Su GY (2020). Functional group dependent screening of organophosphate esters (OPEs) and discovery of an abundant OPE bis-(2-ethylhexyl)-phenyl phosphate in indoor dust. Environ Sci Technol.

[CR43] Mo L, Zheng J, Wang T, Shi YG, Chen BJ, Liu B, Ma YH, Li M, Zhuo L, Chen SJ (2019). Legacy and emerging contaminants in coastal surface sediments around Hainan Island in South China. Chemosphere.

[CR44] Peverly AA, O’Sullivan C, Liu LY, Venier M, Martinez A, Hornbuckle KC, Hites RA (2015). Chicago’s sanitary and ship canal sediment: polycyclic aromatic hydrocarbons, polychlorinated biphenyls, brominated flame retardants, and organophosphate esters. Chemosphere.

[CR45] Pintado-Herrera MG, Wang C, Lu J, Chang YP, Chen W, Li X, Lara-Martín PA (2017). Distribution, mass inventories, and ecological risk assessment of legacy and emerging contaminants in sediments from the Pearl River estuary in China. J Hazard Mater.

[CR46] Schmidt N, Castro-Jiménez J, Fauvelle V, Ourgaud M, Sempéré R (2020). Occurrence of organic plastic additives in surface waters of the Rhône River (France). Environ Pollut.

[CR47] Sibiya I, Poma G, Cuykx M, Covaci A, DasoAdegbenro P, Okonkwo J (2019). Targeted and non-target screening of persistent organic pollutants and organophosphorus flame retardants in leachate and sediment from landfill sites in Gauteng Province, South Africa. Sci Total Environ.

[CR48] Sutton R, Chen D, Sun J, Greig DJ, Wu Y (2019) Characterization of brominated, chlorinated, and phosphate flame retardants in San Francisco Bay, an urban estuary. Sci Total Environ 652:212–223. 10.1016/j.scitotenv.2018.10.096 10.1016/j.scitotenv.2018.10.096 30366322

[CR49] van der Veen I, de Boer J (2012). Phosphorus flame retardants: properties, production, environmental occurrence, toxicity and analysis. Chemosphere.

[CR50] Verbruggen EMJ, Rila JP, Traas TP, Posthuma-Doodeman CJAM, Posthumus R (2005) Environmental risk limits for several phosphate esters, with possible application as flame retardant. http://refhub.elsevier.com/S0160-4120(13)00129-3/rf0285. Accessed 10 May 2019

[CR51] Wang X, He Y, Lin L, Zeng F, Luan T (2014). Application of fully automatic hollow fiber liquid phase microextraction to assess the distribution of organophosphate esters in the Pearl River Estuaries. Sci Total Environ.

[CR52] Wang Y, Kannan P, Halden RU, Kannan K (2019). A nationwide survey of 31 organophosphate esters in sewage sludge from the United States. Sci Total Environ.

[CR53] Wang X, Zhu Q, Yan X, Wang Y, Liao C, Jiang G (2020). A review of organophosphate flame retardants and plasticizers in the environment: analysis, occurrence and risk assessment. Sci Total Environ.

[CR54] Wang Y, Yao YM, Han XX, Li WH, Zhu HK, Wang L, Sun HW, Kannan K (2020). Organophosphate di- and tri-esters in indoor and outdoor dust from China and its implications for human exposure. Sci Total Environ.

[CR55] Wang Z, An C, Chen X, Lee K, Zhang B, Feng Q (2021). Disposable masks release microplastics to the aqueous environment with exacerbation by natural weathering. J Hazard Mater.

[CR56] Wei GL, Li DQ, Zhuo MN, Liao YS, Xie ZY, Guo TL, Li JJ, Zhang SY, Liang ZQ (2015). Organophosphorus flame retardants and plasticizers: sources, occurrence, toxicity and human exposure. Environ Pollut.

[CR57] Wu JY, Zhu T, Chen ZM, Guo JS, Hou XY, Wang DR, Zhang LX, Gao JM (2022). Occurrence, seasonal variation, potential sources, and risks of organophosphate esters in a cold rural area in Northeast China. Sci Total Environ.

[CR58] Xing LQ, Zhang Q, Sun X, Zhu HX, Zhang SH, Xu HZ (2018). Occurrence, distribution and risk assessment of organophosphate esters in surface water and sediment from a shallow freshwater Lake, China. Sci Total Environ.

[CR59] Xu F, Giovanoulis G, van Waes S, Padilla-Sanchez JA, Papadopoulou E, Magnér J, Haug LS, Neels H, Covaci A (2016). Comprehensive study of human external exposure to organophosphate flame retardants via air, dust, and hand wipes: the importance of sampling and assessment strategy. Environ Sci Technol.

[CR60] Yadav IC, Devi NL, Li J, Zhang G, Covaci A (2018). Concentration and spatial distribution of organophosphate esters in the soil-sediment profile of Kathmandu Valley, Nepal: Implication for risk assessment. Sci Total Environ.

[CR61] You J, Chen ZM, Hou XY, Guo JS, Wang CC, Gao JM (2022). Occurrence, potential sources and risks of organophosphate esters in the high-elevation region, Tibet, China. Sci Total Environ.

[CR62] Zeng EY, Tsukada D, Diehl DW, Peng J, Schiff K, Noblet JA, Maruya KA (2005). Distribution and mass inventory of total dichlorobiphenyl dichloroethylene in the water column of the southern California bight. Environ Sci Technol.

[CR63] Zhang Q, Ji C, Yin X, Yan L, Lu M, Zhao M (2016). Thyroid hormone-disrupting activity and ecological risk assessment of phosphorus-containing flame retardants by in vitro, in vivo and in silico approaches. Environ Pollut.

[CR64] Zhang R, Yu K, Li A, Zeng W, Lin T, Wang Y (2020). Occurrence, phase distribution, and bioaccumulation of organophosphate esters (OPEs) in mariculture farms of the Beibu Gulf, China: A health risk assessment through seafood consumption. Environ Pollut.

[CR65] Zhang L, Lu L, Zhu W, Yang B, Lu D, Dan SF, Zhang S (2021). Organophosphorus flame retardants (OPFRs) in the seawater and sediments of the Qinzhou Bay, Northern Beibu Gulf: occurrence, distribution, and ecological risks. Mar Pollut Bull.

[CR66] Zhang Q, Wang YX, Jiang XX, Xu HZ, Luo YQ, Long TT, Li J, Xing L (2021). Spatial occurrence and composition profile of organophosphate esters (OPEs) in farmland soils from different regions of China: implications for human exposure. Environ Pollut.

[CR67] Zhang W, Guo C, Lv J, Li X, Xu J (2022). Organophosphate esters in sediment from Taihu lake, China: Bridging the gap between riverine sources and lake sinks. Front Environ Sci Eng.

[CR68] Zhong MY, Wu HF, Mi WY, Li F, Ji CL, Ebinghaus R, Tang JH, Xie ZY (2018). Occurrences and distribution characteristics of organophosphate ester flame retardants and plasticizers in the sediments of the Bohai and Yellow Seas, China. Sci Total Environ.

[CR69] Zhou Y, Sun J, Wang L, Zhu G, Li M, Liu J, Li Z, Gong H, Wu C, Yin G (2021) Multiple classes of chemical contaminants in soil from an e-waste disposal site in China: Occurrence and spatial distribution. Sci Total Environ 752:141924. 10.1016/j.scitotenv.2020.141924 10.1016/j.scitotenv.2020.141924 32898803

