# Supplementary material for: Occurrence, source apportionment, and ecological risk assessment of organophosphate esters in surface sediment from the Ogun and Osun Rivers, Southwest Nigeria
Source: Environ Sci Pollut Res Int. 2023 Nov 24;30(59):124274–85. doi: 10.1007/s11356-023-31125-z (PMC10746756; doi:10.1007/s11356-023-31125-z)
Supplement: Supplementary file 1 — Supplementary file1 (DOCX 401 KB) [file 11356_2023_31125_MOESM1_ESM.docx]

**Supplementary Information for**

**Occurrence, source apportionment, and ecological risk assessment of organophosphate esters in surface sediment from the Ogun and Osun Rivers, Southwest Nigeria**

Muideen Remilekun Gbadamosi ^a, b^, Adeyemi Lawrence Ogunneye ^b^, David Olaoluwa Jegede ^c^, Mohamed Abou-Elwafa Abdallah ^a^, Stuart Harrad ^a^

^a^ School of Geography, Earth, and Environmental Sciences, University of Birmingham, Birmingham, B15 2TT, UK

^b^ Department of Chemical Sciences, Tai Solarin University of Education, Ijebu-Ode, Ogun State, Nigeria

^c^ Chemistry Unit, Department of Basic Science, Babcock University, Ilishan-Remo, Ogun State, Nigeria

***Author for correspondence (Muideen Gbadamosi)**

**Number of Section: 1**

**Number of Tables: 10**

**Number of Figures: 2**

**Number of Pages: 27**

**Section 1:** **Standards and reagents**

Detailed information about the target OPEs are provided in Table S1. Reference standards of all eight OPEs namely: TCEP, TCIPP, TDCIPP, TPHP, EHDPP, tri-n-buty phosphate (TnBP), tri-m-tolyl phosphate (TMTP) and TBOEP as well as two internal (surrogate) standards (TBP-d_27_ and TPHP-d_15_), and 2,3,4,6-tetrachlorobiphenyl (PCB-62) used as a recovery determination (or syringe) standard (RDS) were purchased from Wellington Laboratories, (Guelph, ON, Canada). The purity of all analytical standards was ˃ 98 %, except TBOEP (˃ 94%). HPLC-grade solvents: acetone (ACE, 99.8 %), ethyl acetate (ETAC, 99.8%), n-hexane (HEX, 95%) and iso-octane (ISOC, 99.5%) were purchased from Fisher Scientific (Loughborough, UK) and Sigma-Aldrich (St Louis, MO, USA). Anhydrous sodium sulfate was purchased from Fisher Scientific (Loughborough, UK) and heated in a furnace for 4 h at 450 ^°^C before use. Hypersep Florisil^®^ SPE cartridges were purchased from Thermo Scientific (Rockwood, USA), with the nitrogen gas used for solvent evaporation purchased from BOC gases, United Kingdom.

**Table S1: Names, abbreviations, formulas, and physicochemical properties of the target OPEs in the present study**

| OPEs classification | Name of target OPEs | Acronym | CAS#. | Boiling Point (°C) | Ρ (mmHg at 25°C) | Molecular weight | Log Koc (kg/L) | Water solubility (mg/L at 25°C) | Log K_OW_ | Density (kg/L) at 25°C |
| --- | --- | --- | --- | --- | --- | --- | --- | --- | --- | --- |
| Cl-OPEs | Tris (2-chloroethyl) phosphate | TCEP | 115-96-8 | 210-220 | 6.13x10^-2^ | 285.49 | 2.48 | 7000 | 1.43 | 1.4 |
|  | Tris(2-chloroisopropyl) Phosphate | TCIPP | 13674-84-5 | 235-248 | 9.23x10^-3^ | 327.56 | 3.05 | 1.6x10^3^ | 2.59 | 1.29 |
|  | Tris (1, 3-dichloro-2-propyl) phosphate | TDCPP | 13674-87-8 | 236-237 | 2.86x10^-7^ | 430.90 | 2.35 | 7 | 3.65 | 1.48 |
| Aryl-OPEs | Triphenyl phosphate | TPHP | 115-86-6 | 370 | 2.00x10^-6^ | 326.28 | 3.72 | 4.59 | 4.70 | 1.2055 |
|  | 2-Ethylhexyl diphenyl phosphate | EHDPP | 1241-94-7 | 232 | 3.3x10^-5^ | 362.40 | 4.21 | 1.9 | 5.73 | 1.090 |
|  | Tri-m-tolyl phosphate | TMTP | 563-04-2 | 260 | 7.8x10^-7^ | 368.4 | 4.1x10^4^ | - | 6.34 | 1.150 |
| Alkyl-OPEs | Tris (butoxyethyl) Phosphate | TBOEP | 78-51-3 | 255 | 1.2x10^-6^ | 398.47 |  | 604 | 3.75 | 13.8 |
|  | Tri-n-butyl phosphate | TnBP | 126-73-8 | 289 | 1.13x10^-3^ | 266.31 | 3.28 | 280 | 4.00 | 0.98 |

*Where: S = solubility (mg/L) in water at 25°C; P = vapor pressure (mmHg) at 25°C; log K_ow_ = n-octanol: water partition coefficient; K*OC=Organic carbon-water partition coefficient & *MW = molecular weight. The data are compiled from databases (Pubchem, 2022) and Pantelaki and Voutsa (2019).*

Table S2. Retention time, quantification and confirmation ions selected for target organophosphate esters (OPEs) and their deuterated IS.

| OPEs | Abbreviation | Retention time (min) | Quantification ion (*m/z*) | Confirmation ion (*m/z*) |
| --- | --- | --- | --- | --- |
| **Deuterated IS** | **d_27_-TnBP** | **8.06** | **103** | **167** |
| Tri-n-butyl phosphate | TnBP | 8.17 | 99 | 155 |
| Tris(2-chloroethyl) phosphate | TCEP | 8.87 | 249 | 251 |
| Tris(2-chloroisopropyl) phosphate | TCIPP | 9.10 | 277 | 279 |
| **Deuterated IS** | **d_15_-TPHP** | **12.69** | **342** | **339** |
| Tris(1,3-dichloro-2-propyl) phosphate | TDCIPP | 12.16 | 380.9 | 382.9 |
| Triphenyl phosphate  Tris(2-butoxyethyl) phosphate | TPHP  TBOEP | 12.75  12.58 | 326  124 | 325  199 |
| 2-Ethylhexyl diphenyl phosphate | EHDPP | 12.99 | 251 | 250 |
| Tri-m-tolyl phosphate | TMTP | 14.37 | 368 | 367 |
| RDS: 2,3,4,6 – Tetrachlorobiphenyl | PCB-62 | 10.09 | 291.9 | 293.9 |

Table S3: Internal standard recovery, blank concentrations, and limit of detection (LOD) and limit of quantification (LOQ) of OPEs.

| Target OPEs | Calibration range (ng/µL) | Linear (R^2^) | Internal standard | Range of IS Recovery (%) (mean ± SD, RSD %) (n=5) | Target OPEs Recovery (%) (n=5) (Spiked at 50 ng/µL) | Procedural Blanks (ng/g) (n=20) | Instrument detection limit (IDL) (LOD) (ng/injection) | LOQ (ng/injection |
| --- | --- | --- | --- | --- | --- | --- | --- | --- |
| TnBP | 0.05-0.75 | 0.999 | d_27_-TnBP | 79.3 – 105 (89.5 ± 10.5, 11.7) | 90.4±8.21 | ND | 7x10^-3^ | 0.95 |
| TCEP | 0.05-0.75 | 0.998 | d_27_-TnBP | 79.3 – 105 (89.5 ± 10.5, 11.7) | 88.4±11.6 | 0.29 ± 0.14 | 3x10^-3^ | 0.43 |
| TCIPP | 0.05-0.75 | 0.997 | d_27_-TnBP | 79.3 – 105 (89.5 ± 10.5, 11.7) | 82.4±7.20 | ND | 3x10^-3^ | 0.38 |
| TDCIPP | 0.05-0.75 | 0.998 | d_15_-TPHP | 72.2 – 88.2 (80.6 ± 6.0, 7.42) | 99.8±6.71 | ND | 5x10^-3^ | 0.57 |
| TPHP | 0.05-0.75 | 0.996 | d_15_-TPHP | 72.2 – 88.2 (80.6 ± 6.0, 7.42) | 100.5±11.6 | ND | 3x10^-3^ | 0.35 |
| EHDPP | 0.05-0.75 | 0.998 | d_15_-TPHP | 72.2 – 88.2 (80.6 ± 6.0, 7.42) | 101.1±12.3 | ND | 2x10^-3^ | 0.28 |
| TBOEP | 0.05-0.75 | 0.999 | d_15_-TPHP | 72.2 – 88.2 (80.6 ± 6.0, 7.42) | 87.9 ± 7.95 | ND | 1.3x10^-2^ | 1.59 |
| TMTP | 0.05-0.75 | 0.982 | d_15_-TPHP | 72.2 – 88.2 (80.6 ± 6.0, 7.42) | 86.3 ± 6.71 | ND | 3x10^-3^ | 0.40 |

Table S4: KMO and Bartlett’s test for the Ogun and Osun River

| **KMO and Bartlett’s Test Ogun River** | | | **Osun River** |
| --- | --- | --- | --- |
| Kaiser-Meyer-Olkin Measure of Sampling Adequacy. | | .769 | 0.716 |
| Bartlett’s Test of Sphericity | Approx. Chi-Square | 123.217 | 37.504 |
|  | Df | 21 | 21 |
|  | Sig. | <.001 | 0.001 |

Table S5: Comparison of the concentrations of OPEs (ng/g dw) in the sediments from the two rivers with previous studies worldwide.

| Study locations and period | TEP | TPRP | TIBP | TNBP | TEHP | TPHP | TMPP | EHDPP | TCEP | TCIPP | TDCIPP | TBOEP | ∑OPEs | Ref |
| --- | --- | --- | --- | --- | --- | --- | --- | --- | --- | --- | --- | --- | --- | --- |
| Ogun River, Nigeria, 2021 | - | - | - | ˂LOQ-LOQ | - | 3.90-121 | - | ˂LOQ-32.1 | 0.74-6.73 | 5.00-46.1 | ˂LOQ-114 | ˂LOQ-1567 | 13.1-2110 | Present study |
| Osun River, Nigeria, 2021 | - | - | - | ˂LOQ-LOQ | - | 0.02-2.61 | - | ˂LOQ-9.85 | 6.71-48.3 | 15.3-70.9 | ˂LOQ-209 | ˂LOQ-245 | 247-589 | Present study |
| Vaal River catchment, South Africa, 2017 | - | - | - | ˂LOQ-166 | ˂LOQ—14.06 | ˂LOQ-6.49 | - | - | ˂LOQ-8.61 | ˂LOQ-4.66 | ˂LOQ-3.35 | 3.53-125.9 | 67.8-278 | Chokwe and Okonkwo, 2019 |
| Guangzhou, South China, 2016-2017 | - | - | - | 0.36-7.5 | 16-3200 | 1.0-29 | - | 0.42-1100 | 0.30-5.8 | 0.30-3.3 | 0.75-47 | 0.47-27 | 17-4400 | Liang et al. 2021 |
| Lake Superior, USA, 2010-2013 | ND-0.39 | NA | 0.63-1.96 | NQ-1.36 | ND | ND-1.04 | - | ND-0.19 | ND-1.90 | ND-1.10 | ND-1.88 | ND-0.46 | 0.80-5.80 | Cao et al. 2017 |
| Lake Michigan, USA, 2010-2013 | ND-NQ | NA | ND-0.59 | ND-1.86 | ND-0.17 | ND-0.91 | - | ND-0.32 | ND-NQ | ND-1.60 | ND-1.99 | ND-1.90 | 0.44-17.6 | Cao et al. 2017 |
| Lake Ontario, USA, 2010-2013 | ND | NA | 0.23-1.07 | 0.22-7.62 | ND-8.38 | ND-9.03 | - | ND-1.44 | ND-NQ | ND-3.37 | ND-NQ | ND-23.7 | 1.38-47.8 | Cao et al. 2017 |
| Sanitary and ship canal, Chicago, USA, 2014 | - | - | - | - | 29-250 | 18-170 | - | 28-690 | 13-44 | 25-190 | - | 320-1600 | - | Peverly et al. 2015 |
| Bagmati River, Nepal, 2014 | - | - | - | 5.04-320 | 657-3020 | 3.33-130 | - | 33.9-418 | 10.9-38.3 | 1.69-892 | 3.89-8.93 | - | 983-7450 | Yadav et al. 2018 |
| Luoma Lake, Fanting and Yi River, China, 2016 | ˂LOD-34.5 | - | - | ˂LOD-0.05 | 0.003-016 | ˂LOD-0.03 | - | ˂LOD-0.14 | 0.01-1.72 | ˂LOD-0.11 | ˂LOD-0.03 | ˂LOD-0.001 | 0.04-35.9 | Xing et al. 2018 |
| Yangtze River Estuary, China, 2017 | ND | ND | - | ND-3.11 | ND-0.623 | ND-2.22 | - | - | 0.113-2.43 | 0.54-17.0 | ND-0.603 | ND-0.305 | 0.977-19.0 | Ji et al. 2022 |
| Beibu Gulf, China, 2020 | n.d.~0.3 | n.d.~2.1 | n.d.~7.2 | n.d.~7.2 | n.d.~<LOQ | n.d.~<LOQ | n.d.~5.4 | n.d.~<LOQ | n.d.~14.5 | n.d.~11.9 | n.d.~0.3 | - | <LOQ~32.2 | Zhang et al. 2021 |
| Pearl River Delta, China, 2010 | n.d.~9.5 | n.d.~2.3 | - | n.d.~37 | 0.9~56 | n.d.~253 | n.d.~11 | n.d.~5.1 | n.d.~58 | n.d.~185 | n.d.~10 | n.d.~46 | 8.3~470 | Tan et al. 2016 |
| Pearl River, South China, 2015 | <MDL~0.52 | 0.21~3.40 | 3.5 | 1.29~12.8 | 0.15~26.7 | 0.42~316.5 | - | <MDL~2.38 | 1.0-26.5 | 2.27~186.3 | <MDL~6.05 | <MDL~19.7 | 13.2~377.1 | Hu et al. 2017 |
| North Pacific and the Arctic Ocean, 2010 | - | - | 0.047~0.55 | 0.06~0.55 | - | n.d.~0.10 | - | - | 0.08~3.90 | 0.94×10^-3^~0.46 | n.d.~0.16 | - | 0.16~4.66 | Ma et al. 2017 |
| Yellow sea and East China sea | <MDL~20.4 | <MDL | - | <MDL~1.11 | 0.08~0.66 | <MDL~0.32 | <MDL~0.075 | <MDL~0.26 | <MDL~5.87 | 0.53~20.3 | <MDL~0.46 | <MDL~1.12 | 1.66~28.7 | Chen et al. 2019 |
| Coast of LOS Angeles, 2007 | n.d.~251 | - | n.d.~251 | n.d.~39.9 | n.d.~176 | n.d.~537 | n.d.~358 | - | n.d.~17.0 | n.d.~13.6 | n.d.~18.5 | n.d.~180 | 0.68~1064 | Li et al. 2019 |
| San Francisco Bay, 2014 | 0.03~0.06 | <0.05 | - | 0.35~1.2 | 2.3-20 | 0.44~7.5 | <0.05 | 0.1~1.5 | 0.03~0.13 | 0.26~1.6 | 0.73~2.0 | 0.51~4.8 | 9.5~33 | Sutton et al. 2019 |
| Bohai and Yellow Seas, China, 2010 | - | - | 0.008~1.11 | 0.004~0.054 | 0.008~3.44 | 0.007~0.21 | - | - | 0.007~0.67 | 0.029~1.52 | 0.002~0.054 | - | 0.083~4.55 | Mi et al. 2019 |
| Laizhou Bay, China, 2017-2018 | 15.0^a^ | 14.2 ^a^ | 11 ^a^ | 33.8 ^a^ | 32.1 ^a^ | 14.2 ^a^ | - | 18.4 ^a^ | 7.4 ^a^ | 16.3 ^a^ | 20.1 ^a^ | 68.2 ^a^ | 304.2 ^a^ | Bekele et al. 2019 |
| Beibu Gulf, 2019 | - | - | 0.6-5.7 | 0.7-4.8 | <MLQ-0.4 | 1.3-5.8 | n.d. | - | 6.1-82.1 | 13.9-92.5 | <MLOQ-1.7 | 5.9-34.5 | 32.9-227 | Zhang et al. 2020 |
| Liao River, North East, China, 2018 | 0.56-11.4 | - | 0.30-12.7 | 2.88-49.1 | 1.36-20.2 | 0.55-6.35 | 0.53-11.1 | 1.19-16.4 | 1.15-30.1 | 0.44-13.3 | 0.48-27.7 | 1.11-69.0 | 19.7-234 | Luo et al. 2020 |
| Bohai Sea, China, 2011-2016 | ND-3.59 | - | ND-125 | ND-25.7 | - | ND-34.5 | ND-7.77 | ND-7.28 | ND-14.8 | ND-39.8 | ND-4.44 | ND-11.9 | 1.76-169 | Liao et al. 2020 |

Note: ND: Not detected; NA: Not available; NQ: Not quantified; MDL: Method detection limit; MLOQ/LOQ: limit of quantitation

Table S6: Concentrations of OPEs (ng/g dw) in sediment samples from the Ogun River

| Sample ID | TnBP | TCEP | TCIPP | TDCIPP | TPHP | EHDPP | TBOEP | TMTP |  |
| --- | --- | --- | --- | --- | --- | --- | --- | --- | --- |
| ARA1 | ˂0.60 | 6.57 | 21.2 | ˂ 0.57 | 16.7 | ˂ 0.28 | ˂ 1.59 | ˂ 0.40 |  |
| ARA2 | ˂0.60 | 6.73 | 21.0 | ˂ 0.57 | 24.7 | 32.1 | ˂ 1.59 | 89.0 |  |
| ARA3 | ˂0.60 | 3.53 | 15.6 | ˂ 0.57 | 14.3 | 23.9 | 1194 | ˂ 0.40 |  |
| ARA4 | ˂0.60 | 5.27 | 15.8 | ˂ 0.57 | 14.3 | 24.3 | 1022 | 121 |  |
| ARA5 | ˂0.60 | 4.50 | 21.7 | 96.6 | 14.4 | 14.9 | 1295 | 104 |  |
| ARA6 | ˂0.60 | 3.41 | 13.6 | ˂ 0.57 | 16.9 | ˂ 0.28 | 1205 | ˂ 0.40 |  |
| ARA7 | ˂0.60 | 1.78 | 10.6 | ˂ 0.57 | 10.1 | 8.55 | 1101 | 51.5 |  |
| ARA8 | ˂0.60 | 3.68 | 13.4 | ˂ 0.57 | 14.4 | 24.5 | 1353 | ˂ 0.40 |  |
| ARA9 | ˂0.60 | 4.30 | 15.3 | ˂ 0.57 | 15.2 | 18.6 | 1313 | 13.2 |  |
| ARA10 | ˂0.60 | 3.71 | 20.6 | 114 | 17.0 | 22.3 | 1563 | 223 |  |
| ARA11 | ˂0.60 | 2.45 | 12.9 | ˂ 0.57 | 13.3 | 29.2 | 1227 | ˂ 0.40 |  |
| ARA12 | ˂0.60 | 3.34 | 14.2 | 48.8 | 6.78 | 11.1 | ˂ 1.59 | ˂ 0.40 |  |
| ARA13 | ˂0.60 | 2.05 | 10.5 | ˂ 0.57 | 9.57 | 10.4 | 1089 | ˂ 0.40 |  |
| ARA14 | ˂0.60 | 6.17 | 20.2 | ˂ 0.57 | 17.3 | 16.6 | 1197 | ˂ 0.40 |  |
| ARA15 | ˂0.60 | 2.04 | 9.0 | ˂ 0.57 | 13.6 | 13.8 | 1567 | 121 |  |
| ARA16 | ˂0.60 | 3.16 | 17.3 | ˂ 0.57 | 18.3 | 29.2 | 1440 | ˂ 0.40 |  |
| ARA17 | ˂0.60 | 3.55 | 17.3 | 12.04 | 8.9 | 13.8 | 1377 | ˂ 0.40 |  |
| ARA18 | ˂0.60 | 2.47 | 13.0 | ˂ 0.57 | 13.5 | 16.8 | 1181 | 115 |  |
| ARA19 | ˂0.60 | 2.05 | 11.0 | ˂ 0.57 | 8.92 | 8.95 | 899 | 27.4 |  |
| ARA20 | ˂0.60 | 3.02 | 12.1 | ˂ 0.57 | 6.38 | 13.7 | 678 | ˂ 0.40 |  |
| IRO1 | ˂0.60 | 4.45 | 12.4 | ˂ 0.57 | 121 | 22.4 | 48.8 | 166 |  |
| IRO2 | ˂0.60 | 2.66 | 10.9 | 14.92 | 21.5 | 28.0 | 1260 | 26.7 |  |
| IRO3 | ˂0.60 | 2.51 | 8.54 | ˂ 0.57 | 14.7 | 24.1 | 1104 | ˂ 0.40 |  |
| IRO4 | ˂0.60 | 1.48 | 7.85 | ˂ 0.57 | 5.75 | ˂ 0.28 | 1503 | 67.8 |  |
| IRO5 | ˂0.60 | 3.10 | 12.4 | 83.6 | 11.7 | 11.9 | 1084 | 12.8 |  |
| IRO6 | ˂0.60 | 2.09 | 8.04 | 7.96 | 9.90 | 14.6 | 961 | 23.0 |  |
| IRO7 | ˂0.60 | 2.07 | 9.22 | 3.22 | 7.70 | 11.1 | 634 | 64.4 |  |
| IRO8 | ˂0.60 | 2.08 | 6.46 | 7.78 | 10.3 | 9.34 | 755 | ˂ 0.40 |  |
| IRO9 | ˂0.60 | 0.97 | 5.03 | 2.94 | 4.27 | 4.48 | 369 | 29.4 |  |
| IRO10 | ˂0.60 | 1.59 | 10.2 | 7.56 | 5.78 | 7.22 | 390 | 0.40 |  |
| IRO11 | ˂0.60 | 2.09 | 8.46 | 7.04 | 7.73 | 10.3 | 228 | 63.8 |  |
| IR012 | ˂0.60 | 5.41 | 12.7 | ˂ 0.57 | 16.5 | 21.4 | 1215 | 153 |  |
| IRO13 | ˂0.60 | 0.74 | 5.17 | 5.13 | 3.91 | 5.17 | 390 | 9.56 |  |
| IRO14 | ˂0.60 | 2.60 | 8.22 | ˂ 0.57 | 9.49 | 16.4 | 993 | ˂ 0.40 |  |
| IRO15 | ˂0.60 | 2.07 | 10.3 | 4.76 | 6.36 | 10.3 | ˂ 1.59 | 67.5 |  |
| IRO16 | ˂0.60 | 1.49 | 5.72 | ˂ 0.57 | 4.75 | 6.04 | 175 | ˂ 0.40 |  |
| IRO17 | ˂0.60 | 2.17 | 9.36 | ˂ 0.57 | 6.54 | 14.3 | ˂ 1.59 | 124 |  |
| IRO18 | ˂0.60 | 2.62 | 12.6 | ˂ 0.57 | 7.55 | 10.4 | 306 | ˂ 0.40 |  |
| IRO19 | ˂0.60 | 3.23 | 14.8 | 8.61 | 9.94 | 10.6 | 226 | ˂ 0.40 |  |
| IRO20 | ˂0.60 | 2.31 | 11.4 | ˂ 0.57 | 14.0 | 13.0 | 396 | ˂ 0.40 |  |
| KARA_1 | ˂ 0.60 | 3.78 | 13.5 | 8.09 | 9.02 | 12.1 | 36.7 | 142 |  |
| KARA_2 | ˂ 0.60 | 6.55 | 46.1 | ˂ 0.57 | 21.3 | ˂ 0.28 | 124 | ˂ 0.40 |  |
| KARA_3 | ˂ 0.60 | 4.25 | 14.2 | 10.7 | 16.5 | 13.9 | 77.3 | ˂ 0.40 |  |
| KARA_4 | ˂ 0.60 | 4.17 | 15.1 | 10.5 | 11.5 | ˂ 0.28 | 158 | ˂ 0.40 |  |
| KARA_5 | ˂ 0.60 | 1.02 | 5.72 | 3.45 | 4.36 | 8.70 | 35.2 | ˂ 0.40 |  |
| KARA_6 | ˂ 0.60 | 3.31 | 12.1 | ˂ 0.57 | 10.4 | 19.1 | 25.6 | ˂ 0.40 |  |
| KARA_7 | ˂ 0.60 | 5.99 | 14.3 | ˂ 0.57 | 14.5 | 21.0 | 75.1 | 148 |  |
| KARA_8 | ˂ 0.60 | 3.09 | 17.4 | ˂ 0.57 | 16.8 | 19.0 | 24.7 | 89.2 |  |
| KARA_9 | ˂ 0.60 | 2.39 | 11.0 | ˂ 0.57 | 8.42 | 16.5 | 68.6 | 172 |  |
| KARA_10 | ˂ 0.60 | 1.77 | 10.1 | ˂ 0.57 | 5.68 | 9.51 | 22.3 | 124 |  |
| KARA_11 | ˂ 0.60 | 3.83 | 14.1 | 9.66 | 11.8 | 20.7 | 24.1 | ˂ 0.40 |  |
| KARA_12 | ˂ 0.60 | 2.31 | 13.5 | 11.5 | 9.57 | 17.0 | 86.0 | ˂ 0.40 |  |
| KARA_13 | ˂ 0.60 | 2.51 | 13.0 | ˂ 0.57 | 8.23 | 6.13 | 59.7 | ˂ 0.40 |  |
| KARA_14 | ˂ 0.60 | 3.96 | 14.9 | 10.6 | 10.1 | 15.1 | 24.8 | ˂ 0.40 |  |
| KARA_15 | ˂ 0.60 | 3.78 | 23.1 | ˂ 0.57 | 15.9 | 23.2 | 35.8 | ˂ 0.40 |  |
| KARA_16 | ˂ 0.60 | 2.71 | 11.3 | 5.40 | 5.74 | 8.63 | 25.6 | 86.6 |  |
| KARA_17 | ˂ 0.60 | 2.63 | 9.84 | 6.61 | 11.2 | 12.1 | 25.2 | 77.2 |  |
| KARA_18 | ˂ 0.60 | 1.97 | 7.71 | ˂ 0.57 | 8.11 | 12.6 | 28.6 | ˂ 0.40 |  |
| KARA_19 | ˂ 0.60 | 2.38 | 10.4 | 26.5 | 10.4 | 15.2 | 13.5 | ˂ 0.40 |  |
| KARA_20 | ˂ 0.60 | 2.18 | 9.66 | ˂ 0.57 | 10.4 | 14.8 | 50.1 | 94.0 | **ΣOPEs** |
| **Mean** | **˂ 0.60** | **3.10** | **13.1** | **9.12** | **13.2** | **14.2** | **563** | **43.7** | 660 |
| **Median** | **˂ 0.60** | **2.64** | **12.4** | **0.57** | **10.4** | **13.8** | **337** | **0.40** | 378 |
| **SD** |  | **1.41** | **6.0** | **22.1** | **14.9** | **7.60** | **560** | **58.8** | 671 |
| **95^th^ Percentile** | **˂ 0.60** | **6.18** | **21.2** | **50.5** | **21.3** | **28.0** | **1443** | **153** | 1724 |
| **Min** | **˂ 0.60** | **0.74** | **5.0** | **0.57** | **3.9** | **0.28** | **1.59** | **0.40** | 13.1 |
| **Max** | **˂ 0.60** | **6.73** | **46.1** | **114** | **121** | **32.1** | **1567** | **223** | 2110 |
| **DF (%)** | **0** | **100** | **100** | **42** | **100** | **92** | **92** | **48** | - |

Table S7: Concentrations of OPEs (ng/g dw) in sediment samples from Osun River

| Sample ID | TnBP | TCEP | TCIPP | TDCIPP | TPHP | EHDPP | TBOEP | TMTP |  |
| --- | --- | --- | --- | --- | --- | --- | --- | --- | --- |
| OSSED1 | ˂0.60 | 27.5 | 25.8 | ˂ 0.57 | 0.39 | ˂ 0.28 | 128 | 1.14 |  |
| OSSED2 | ˂0.60 | 20.7 | 22.9 | ˂ 0.57 | 1.29 | ˂ 0.28 | ˂ 1.59 | 0.60 |  |
| OSSED3 | ˂0.60 | 22.9 | 24.2 | 0.02 | 1.14 | 9.85 | ˂ 1.59 | 1.34 |  |
| OSSED4 | ˂0.60 | 32.9 | 25.2 | ˂ 0.57 | 0.02 | ˂ 0.28 | ˂ 1.59 | 0.10 |  |
| OSSED5 | ˂0.60 | 48.3 | 70.9 | 6.65 | 0.83 | ˂ 0.28 | ˂ 1.59 | ˂0.40 |  |
| OSSED6 | ˂0.60 | 19.5 | 22.0 | ˂ 0.57 | 0.53 | 1.96 | 176 | ˂0.40 |  |
| OSSED7 | ˂0.60 | 22.6 | 24.3 | 19.2 | 0.50 | 2.48 | 245 | 1.75 |  |
| OSSED8 | ˂0.60 | 22.4 | 23.9 | 0.59 | 0.49 | 2.15 | 133 | 0.42 |  |
| OSSED9 | ˂0.60 | 16.8 | 22.2 | ˂ 0.57 | 0.53 | 2.07 | 130 | ˂0.40 |  |
| OSSED10 | ˂0.60 | 20.1 | 31.0 | ˂ 0.57 | 0.58 | 3.19 | 130 | ˂0.40 |  |
| OSSED11 | ˂0.60 | 22.0 | 28.9 | 41.8 | 0.54 | 2.65 | 136 | ˂0.40 |  |
| OSSED12 | ˂0.60 | 32.1 | 33.6 | 209 | 0.54 | 2.89 | 168 | 2.15 |  |
| OSSED13 | ˂0.60 | 20.2 | 29.0 | ˂ 0.57 | 0.49 | 2.07 | 146 | 1.90 |  |
| OSSED14 | ˂0.60 | 6.71 | 15.3 | ˂ 0.57 | 0.51 | 2.54 | 125 | 1.88 |  |
| OSSED15 | ˂0.60 | 22.2 | 20.0 | ˂ 0.57 | 0.58 | 3.19 | 130 | ˂0.40 |  |
| OSSED16 | ˂0.60 | 13.1 | 15.4 | 0.56 | 2.61 | 2.45 | 55.8 | 1.56 |  |
| OSSED17 | ˂0.60 | 11.9 | 21.6 | 2.05 | 1.67 | 2.34 | 80.0 | 0.23 |  |
| OSSED18 | ˂0.60 | 18.8 | 17.3 | ˂ 0.57 | 1.21 | ˂ 0.28 | 66.2 | ˂0.40 |  |
| OSSED19 | ˂0.60 | 13.8 | 16.7 | 8.21 | 2.29 | 1.71 | 123 | ˂0.40 |  |
| OSSED20 | ˂0.60 | 14.6 | 18.1 | ˂ 0.57 | 2.22 | 1.74 | 89.4 | ˂0.40 | **ΣOPEs** |
| **Mean** | **˂ 0.60** | **21.5** | **25.4** | **14.7** | **0.95** | **2.24** | **103** | **0.83** | 169 |
| **Median** | **˂ 0.60** | **20.4** | **23.4** | **0.57** | **0.56** | **2.11** | **126** | **0.40** | 174 |
| **SD** |  | **8.97** | **11.8** | **46.8** | **0.72** | **2.06** | **65.9** | **0.67** | 137 |
| **95^th^ Percentile** | **˂ 0.60** | **33.6** | **35.5** | **50.1** | **2.31** | **3.53** | **179** | **1.92** | 307 |
| **Min** | **˂ 0.60** | **6.71** | **15.3** | **0.02** | **0.02** | **0.28** | **1.59** | **0.10** | 24.7 |
| **Max** | **˂ 0.60** | **48.3** | **70.9** | **209** | **2.61** | **9.85** | **245** | **2.15** | 589 |
| **DF (%)** | **0** | **100** | **100** | **45** | **100** | **75** | **80** | **55** | - |

Table S8a: Pearson correlation coefficients between concentrations of target OPEs in sediment samples from the Ogun River

| **Correlations** | | | | | | | | |
| --- | --- | --- | --- | --- | --- | --- | --- | --- |
| OPES |  | TCEP | TCIPP | TDCIPP | TPHP | EHDPP | TBOEP | TMTP |
| TCEP | Pearson Correlation | 1 | .850^**^ | -.040 | .696^**^ | .038 | -.116 | -.042 |
|  | Sig. (2-tailed) |  | <.001 | .762 | <.001 | .773 | .379 | .749 |
| TCIPP | Pearson Correlation |  | 1 | .008 | .581^**^ | -.061 | -.084 | -.104 |
|  | Sig. (2-tailed) |  |  | .951 | <.001 | .642 | .524 | .429 |
| TDCIPP | Pearson Correlation |  |  | 1 | -.151 | .054 | -.057 | .021 |
|  | Sig. (2-tailed) |  |  |  | .250 | .679 | .664 | .874 |
| TPHP | Pearson Correlation |  |  |  | 1 | .159 | .093 | .084 |
|  | Sig. (2-tailed) |  |  |  |  | .226 | .480 | .523 |
| EHDPP | Pearson Correlation |  |  |  |  | 1 | .055 | .169 |
|  | Sig. (2-tailed) |  |  |  |  |  | .679 | .197 |
| TBOEP | Pearson Correlation |  |  |  |  |  | 1 | -.038 |
|  | Sig. (2-tailed) |  |  |  |  |  |  | .770 |
| TMTP | Pearson Correlation |  |  |  |  |  |  | 1 |
|  | Sig. (2-tailed) |  |  |  |  |  |  |  |
| **. Correlation is significant at the 0.01 level (2-tailed). | | | | | | | | |

Table S8b: Pearson correlation coefficients between concentrations of target OPEs in sediment samples from the Osun River

| **Correlations** | | | | | | | | |
| --- | --- | --- | --- | --- | --- | --- | --- | --- |
| OPEs |  | TCEP | TCIPP | TDCIPP | TPHP | EHDPP | TBOEP | TMTP |
| TCEP | Pearson Correlation | 1 | .790^**^ | .232 | -.428 | -.382 | -.419 | -.159 |
|  | Sig. (2-tailed) |  | <.001 | .324 | .060 | .097 | .066 | .503 |
| TCIPP | Pearson Correlation |  | 1 | .347 | -.263 | -.232 | -.346 | -.050 |
|  | Sig. (2-tailed) |  |  | .134 | .263 | .326 | .135 | .835 |
| TDCIPP | Pearson Correlation |  |  | 1 | -.018 | -.045 | .337 | .093 |
|  | Sig. (2-tailed) |  |  |  | .939 | .852 | .146 | .698 |
| TPHP | Pearson Correlation |  |  |  | 1 | .271 | .177 | .280 |
|  | Sig. (2-tailed) |  |  |  |  | .248 | .455 | .232 |
| EHDPP | Pearson Correlation |  |  |  |  | 1 | .407 | .356 |
|  | Sig. (2-tailed) |  |  |  |  |  | .075 | .123 |
| TBOEP | Pearson Correlation |  |  |  |  |  | 1 | .257 |
|  | Sig. (2-tailed) |  |  |  |  |  |  | .273 |
| TMTP | Pearson Correlation |  |  |  |  |  |  | 1 |
|  | Sig. (2-tailed) |  |  |  |  |  |  |  |
| **. Correlation is significant at the 0.01 level (2-tailed). | | | | | | | | |

Table S9a: Principal component analysis (after varimax rotation) showing contribution of statistically significant variables (in bold) for the Ogun River

| **Rotated Component Matrix^a^** | | | | |  |
| --- | --- | --- | --- | --- | --- |
|  | Component | | | |  |
|  | 1 | 2 | 3 | 4 | Communalities |
| TCEP | **0.950** | -0.019 | -0.098 | 0.009 | 0.913 |
| TCIPP | **0.909** | -0.154 | -0.093 | 0.068 | 0.864 |
| TPHP | **0.829** | 0.217 | 0.167 | -0.170 | 0.791 |
| TMTP | -0.065 | **0.792** | -0.241 | -0.135 | 0.708 |
| EHDPP | 0.076 | **0.721** | 0.274 | 0.199 | 0.641 |
| TBOEP | -0.046 | -0.008 | **0.937** | -0.066 | 0.884 |
| TDCIPP | -0.047 | 0.032 | -0.060 | **0.966** | 0.941 |
| Eigen-value | 2.430 | 1.220 | 1.060 | 1.030 |  |
| % of variance explained | 34.721 | 17.431 | 15.143 | 14.711 |  |
| Cumulative (%) | 34.721 | 52.152 | 67.295 | 82.007 |  |

Table S9b: Principal component analysis (after varimax rotation) showing contribution of statistically significant variables (in bold) for the Osun River

| **Rotated Component Matrix^a^** | | | |
| --- | --- | --- | --- |
|  | Component | | |
|  | 1 | 2 | 3 |
| TCIPP | **.926** | -.066 | .083 |
| TCEP | **.887** | -.277 | -.013 |
| TMTP | .108 | **.838** | .115 |
| TPHP | -.274 | **.642** | -.084 |
| EHDPP | -.299 | **.640** | .180 |
| TDCIPP | .350 | -.006 | **.842** |
| TBOEP | -.468 | .232 | **.762** |
| Eigen-value | 2.162 | 1.658 | 1.349 |
| % of variance explained | 30.883 | 26.681 | 20.278 |
| Cumulative (%) | 30.883 | 57.564 | 77.842 |

Table S10: RQ values in Ogun and Osun River Sediments

|  | | | | | | | Present study | | | | | | | |  |
| --- | --- | --- | --- | --- | --- | --- | --- | --- | --- | --- | --- | --- | --- | --- | --- |
|  |  |  |  |  |  |  | Median (MEC) | | Median RQ | | 95^th^ percentile (MEC) | | 95^th^ Percentile RQ | |  |
| OPEs | Biota | Endpoint | Exposure time | Toxic effect | PNEC sediment (ng/g dw) | RQ max (sediment) | **Ogun River** | **Osun River** | **Ogun River** | **Osun River** | **Ogun River** | **Osun River** | **Ogun River** | **Osun River** | Reference |
| TCEP | Zebrafish | LC50 | 96 hr | mortality | 6100 | 0.03 | 2.64 | 20.5 | 0.0004 | 0.0034 | 6.18 | 33.6 | 0.0010 | 0.0055 | Du et al. 2015 |
|  | Daphnia magna | EC50 | 48 hr | mortality | 11501 | 0.01 | 2.64 | 20.5 | 0.0002 | 0.0018 | 6.18 | 33.6 | 0.0005 | 0.0029 | Cristale et al. 2013 |
|  | Scededesmus subspicatus | EC10 | 48 hr | growth rate | 1963 | 0.08 | 2.64 | 20.5 | 0.0013 | 0.0104 | 6.18 | 33.6 | 0.0031 | 0.0171 | European commission, 2009 |
| TCIPP | Zebrafish | LC50 | 96 hr | mortality | 219 | 5.94 | 12.4 | 23.4 | 0.0566 | 0.1068 | 21.2 | 35.5 | 0.0968 | 0.1621 | Du et al. 2015 |
|  | Daphnia magna | EC50 | 48 hr | mortality | 1318 | 0.99 | 12.4 | 23.4 | 0.0094 | 0.0178 | 21.2 | 35.5 | 0.0161 | 0.0269 | Cristale et al. 2013 |
|  | Daphnia magna | NOEC | 21 d | reproduction | 10380 | 0.13 | 12.4 | 23.4 | 0.0012 | 0.0023 | 21.2 | 35.5 | 0.0020 | 0.0034 | European commission, 2009 |
| TDCIPP | Zebrafish | LC50 | 96 hr | mortality | 9.4 | 2.03 | 0.57 | 0.57 | 0.0606 | 0.0606 | 50.5 | 50.2 | 5.3723 | 5.3404 | Du et al. 2015 |
|  | Zebrafish | LC50 | 120 hr | mortality | 157 | 0.12 | 0.57 | 0.57 | 0.0036 | 0.0036 | 50.5 | 50.2 | 0.3217 | 0.3197 | Liu et al. 2013a |
|  | Zebrafish | LC50 | 90 hr | mortality | 82 | 0.23 | 0.57 | 0.57 | 0.0070 | 0.0070 | 50.5 | 50.2 | 0.6159 | 0.6122 | McGee et al. 2012 |
|  | Daphnia magna | EC50 | 48 hr | mortality | 174 | 0.11 | 0.57 | 0.57 | 0.0033 | 0.0033 | 50.5 | 50.2 | 0.2902 | 0.2885 | Cristale et al. 2013 |
|  | Zebrafish | LOEC | 5 d | mortality & malformations | 482 | 0.04 | 0.57 | 0.57 | 0.0012 | 0.0012 | 50.5 | 50.2 | 0.1048 | 0.1041 | Dishaw et al. 2014 |
|  | Daphnia magna | NOEC | 21 d | reproduction | 224 | 0.09 | 0.57 | 0.57 | 0.0025 | 0.0025 | 50.5 | 50.2 | 0.2254 | 0.2241 | European commission, 2009 |
| TPHP | Zebrafish | LC50 | 96 hr | mortality | 538 | 0.47 | 10.4 | 0.56 | 0.0193 | 0.0010 | 21.3 | 2.31 | 0.0396 | 0.0043 | Du et al. 2015 |
|  | Zebrafish | LC50 | 96 hr | mortality | 803 | 0.32 | 10.4 | 0.56 | 0.0130 | 0.0007 | 21.3 | 2.31 | 0.0265 | 0.0029 | Du et al. 2015 |
|  | Zebrafish | LC50 | 120 hr | mortality | 15534 | 0.02 | 10.4 | 0.56 | 0.0007 | 0.0000 | 21.3 | 2.31 | 0.0014 | 0.0001 | Liu et al. 2013a |
|  | Daphnia magna | EC50 | 48 hr | mortality | 890 | 0.28 | 10.4 | 0.56 | 0.0117 | 0.0006 | 21.3 | 2.31 | 0.0239 | 0.0026 | Cristale et al. 2013 |
|  | Zebrafish | NOEC | 21 d | reproduction | 210 | 1.2 | 10.4 | 0.56 | 0.0495 | 0.0027 | 21.3 | 2.31 | 0.1014 | 0.0110 | Liu et al. 2013b |
|  | Ankistrodesmus falcatus | EC10 | 5 d | growth rate | 84 | 3.0 | 10.4 | 0.56 | 0.1238 | 0.0067 | 21.3 | 2.31 | 0.2536 | 0.0275 | Verbruggen et al. 2005 |
| EHDPP | Daphnia magna | EC50 | 48 hr | mortality | 503 | 0.57 | 13.8 | 2.11 | 0.0274 | 0.0042 | 28 | 3.53 | 0.0557 | 0.0070 | Cristale et al. 2013 |
| TBOEP | Zebrafish | LC50 | 96 hr | mortality | 8012 | 0.02 | 337 | 126 | 0.0421 | 0.0157 | 1442 | 179 | 0.1800 | 0.0223 | Du et al. 2015 |
|  | Zebrafish | LC50 | 120 hr | mortality | 25668 | 0.005 | 337 | 126 | 0.0131 | 0.0049 | 1442 | 179 | 0.0562 | 0.0070 | Han et al. 2014 |
|  | Daphnia magna | EC50 | 48 hr | mortality | 91156 | 0.001 | 337 | 126 | 0.0037 | 0.0014 | 1442 | 179 | 0.0158 | 0.0020 | Cristale et al. 2013 |
|  | Pimephales promelas | LC50 | 96 hr | mortality | 31185 | 0.004 | 337 | 126 | 0.0108 | 0.0040 | 1442 | 179 | 0.0462 | 0.0057 | Verbruggen et al. 2005 |
| TnBP | Zebrafish | LC50 | 96 hr | mortality | 1490 | 0.04 | 0.6 | 0.6 | 0.0004 | 0.0004 | 0.6 | 0.6 | 0.0004 | 0.0004 | Du et al. 2015 |
|  | Daphnia magna | EC50 | 48 hr | mortality | 2287 | 0.03 | 0.6 | 0.6 | 0.0003 | 0.0003 | 0.6 | 0.6 | 0.0003 | 0.0003 | Cristale et al. 2013 |
|  | Scededesmus subspicatus | EC10 | 48 hr | growth rate | 12576 | 0.005 | 0.6 | 0.6 | 0.0000 | 0.0000 | 0.6 | 0.6 | 0.0000 | 0.0000 | Verbruggen et al. 2005 |
|  | Phaeodactylum tricornutum | EC50 | 72 hr | growth rate | 42 | 1.5 | 0.6 | 0.6 | 0.0143 | 0.0143 | 0.6 | 0.6 | 0.0143 | 0.0143 | Liu et al. 2019 |
| TMTP | - | - | - | - | - | - | 0.4 | 0.4 |  |  | 153 | 1.92 | - | - |  |

NB: Values highlighted in red indicate OPEs posing moderate or high ecological risk.


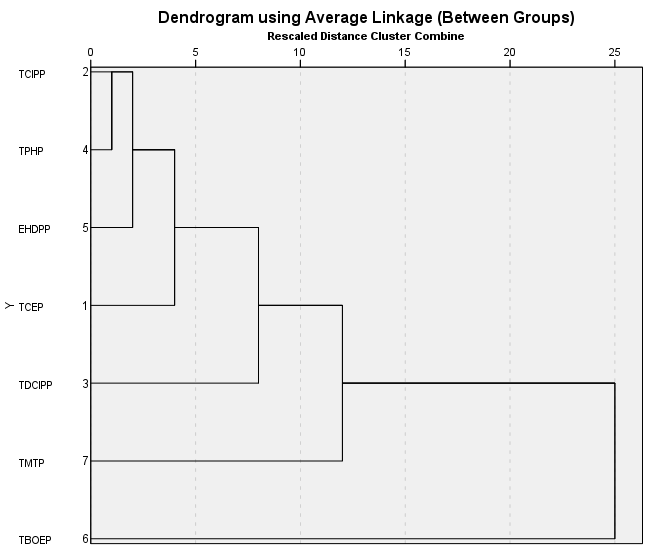


Fig. S1a: Dendrogram showing the cluster formation of OPEs in sediment samples from the Ogun River


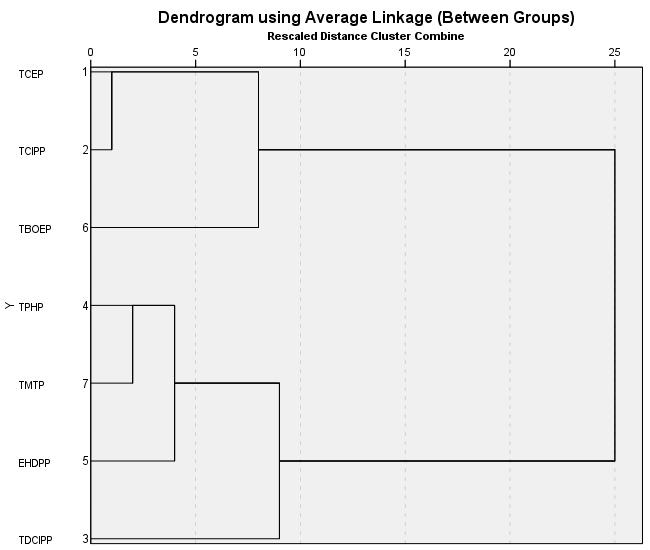


Fig. S1b: Dendrogram showing the cluster formation of OPEs in sediment samples from the Osun River

Fig. S2: Comparison of median OPEs concentrations (ng/g dw) in sediment from the Ogun and Osun rivers


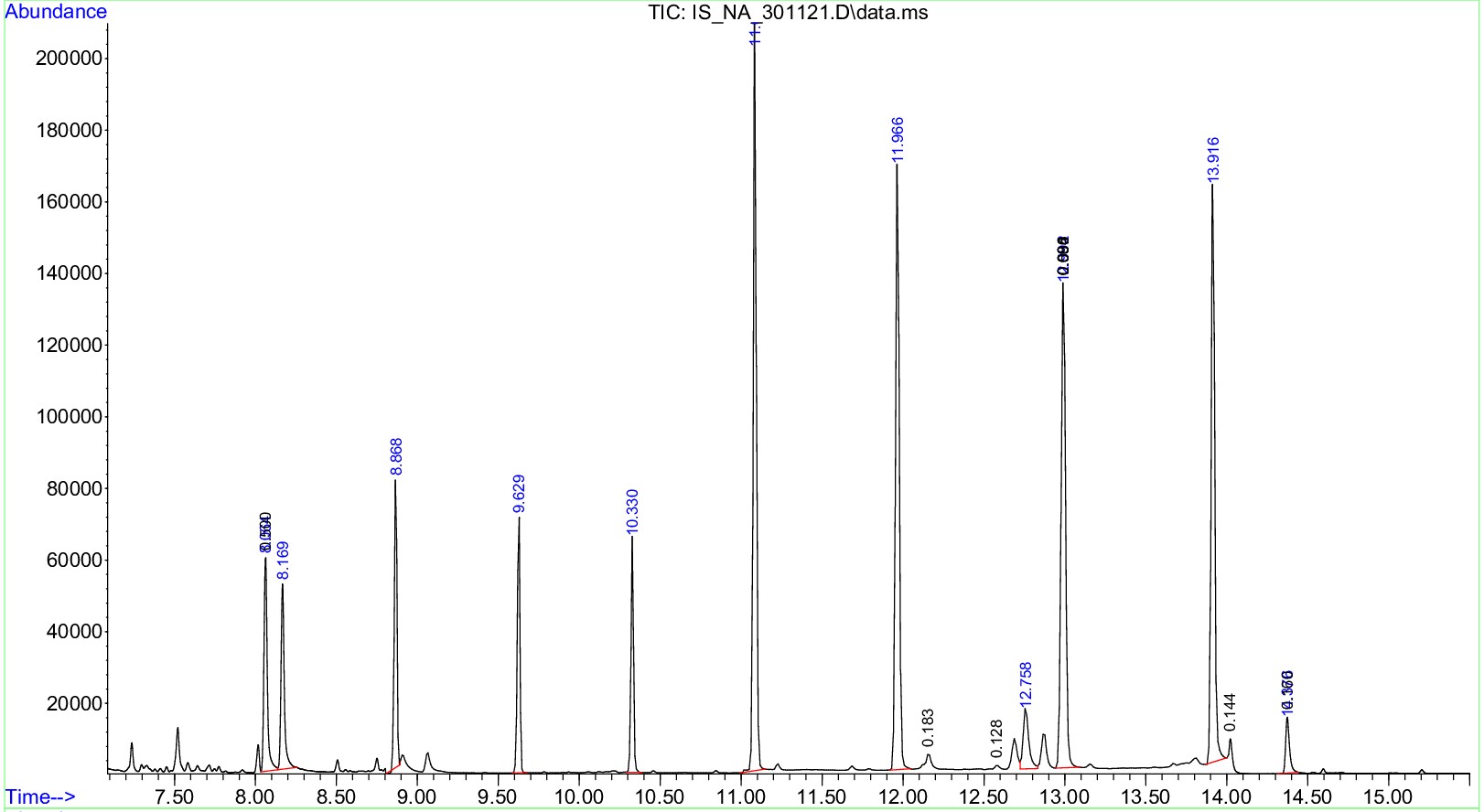


Fig. S3: Internal standard chromatogram


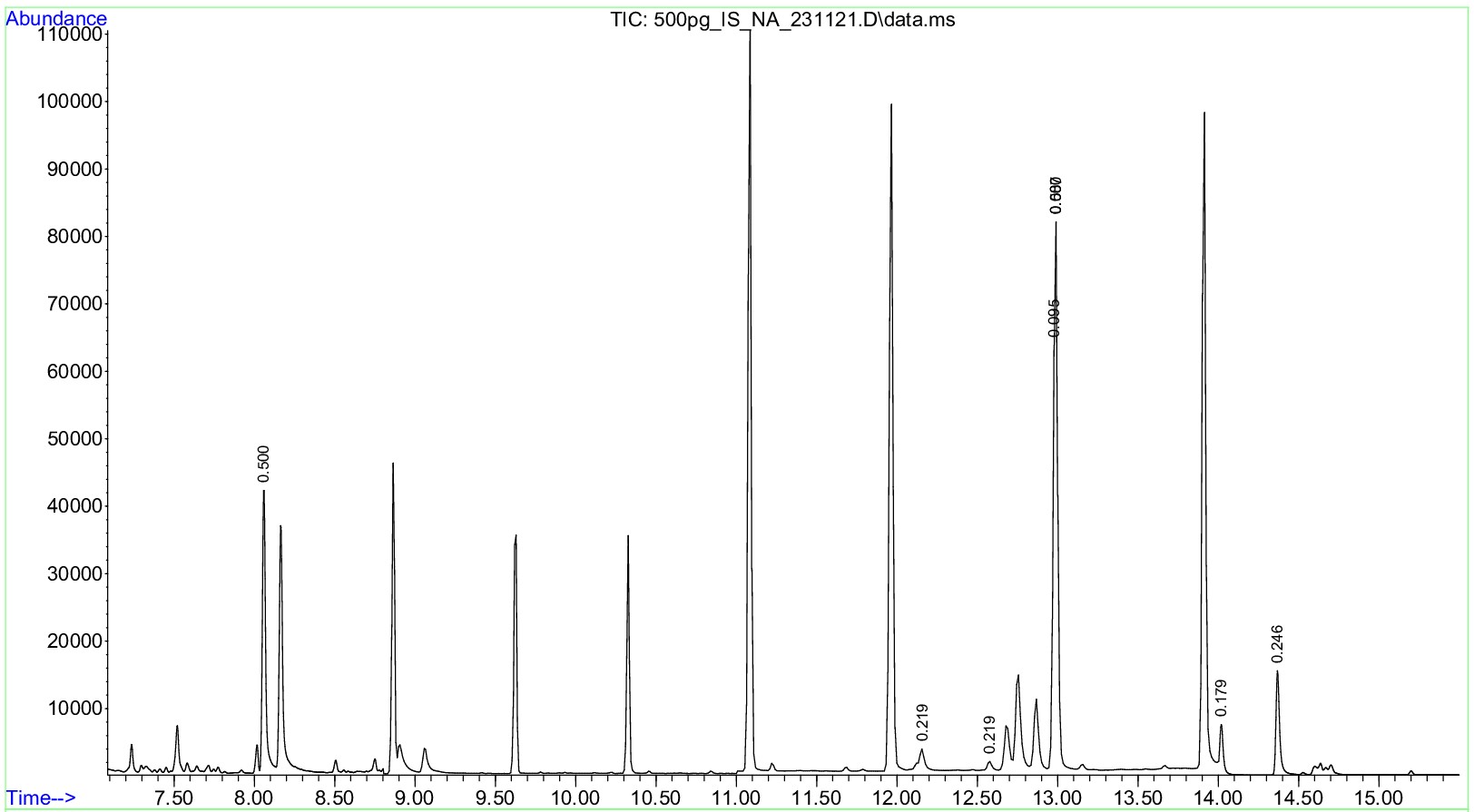


Fig. S4: Internal standard chromatogram

**References**

Bekele TG, Zhao H, Wang Q, Chen J (2019) Bioaccumulation and Trophic Transfer of Emerging Organophosphate Flame Retardants in the Marine Food Webs of Laizhou Bay, North China. Environ. Sci. Technol. 53, 13417-13426.

Cao D, Guo J, Wang Y, Li Z, Liang K, Corcoran MB, Hosseini S, Bonina SMC, Rockne KJ, Sturchio NC, Giesy JP, Liu J, Li A, Jiang G (2017) Organophosphate esters in sediment of the Great Lakes. Environ. Sci. Technol. 51 (3), 1441–1449.

Chen M, Gan Z, Qu B, Chen S, Dai Y, Bao X (2019) Temporal and seasonal variation and ecological risk evaluation of flame retardants in seawater and sediments from Bohai Bay near Tianjin, China during 2014 to 2017. Mar. Pollut. Bull. 146, 874-883.

Chokwe TB, Okonkwo JO (2019) Occurrence, distribution and ecological risk assessment of organophosphorus flame retardants and plasticizers in sediment samples along the Vaal River catchment, South Africa. Emerg. Contam. 5, 173–178. <https://doi.org/10.1016/j.emcon.2019.05.003>.

Cristale J, García VA, Barata C, Lacorte S (2013) Priority and emerging flame retardants in rivers: occurrence in water and sediment, Daphnia magna toxicity and risk assessment. Environ. Int. 59 (3), 232–243. <https://doi.org/10.1016/j.envint.2013.06.011>.

Dishaw LV, Hunter DL, Padnos B, Padilla S, Stapleton HM (2014) Developmental exposure to organophosphate flame retardants elicits overt toxicity and alters behavior in early life stage zebrafish (Danio rerio). Toxicol. Sci. 142 (2), 445–454. <https://doi>. org/10.1093/toxsci/kfu194.

Du ZK, Wang G.W, Gao SX, Wang ZY (2015) Aryl organophosphate flame retardants induced cardiotoxicity during zebrafish embryogenesis: by disturbing expression of the transcriptional regulators. Aquat. Toxicol. 161, 25–32. <https://doi.org/10.1016/j>. aquatox.2015.01.027.

European Commission (2009) European Union risk assessment report: tris (2-chloroethyl) phosphate, TCEP. <https://echa.europa.eu/documents/10162/2663989d-1795-44a1-> 8f50-153a81133258, Accessed date: 25/06/22.

Han Z, Wang Q, Fu J, Chen H, Zhao Y, Zhou B, Gong Z, Wei S, Li J, Liu H, Zhang X, Liu C, Yu H (2014) Multiple bio-analytical methods to reveal possible molecular mechanisms of developmental toxicity in zebrafish embryos/larvae exposed to tris (2-butoxyethyl) phosphate. Aquat. Toxicol. 150, 175–181. <https://doi.org/10.1016/j>. aquatox.2014.03.013.

Hu YX, Sun YX, Li X, Xu WH, Zhang Y, Luo XJ, Dai SH, Xu XR, Mai BX (2017) Organophosphorus flame retardants in mangrove sediments from the Pearl River Estuary, South China. Chemosphere, 181, 433-439.

Ji B, Liu Y, Wu Y, Liang Y, Gao S, Zeng X, Yao P, Yu Z (2022) Organophosphate esters and synthetic musks in the sediments of the Yangtze River Estuary and adjacent East China Sea: Occurrence, distribution, and potential ecological risks. Mar Pollut Bull. 179:113661. doi: 10.1016/j.marpolbul.2022.113661.

Li J, Wang J, Taylor AR, Cryder Z, Schlenk D, Gan J (2019) Inference of Organophosphate Ester Emission History from Marine Sediment Cores Impacted by Wastewater Effluents. Environ. Sci. Technol. 53, 8767-8775.

Liang C, Peng B, Wei G-L, Gong Y, Liu G, Zeng L, Liu L-Y, Zeng EY (2021) Organophosphate diesters in urban River sediment from South China: call for more research on their occurrence and fate in field environment. ACS EST Water 1, 871–880

Liao C, Kim U-J, Kannan K (2020) Occurrence and distribution of organophosphate esters in sediment from northern Chinese coastal waters. Sci. Total Environ. 704, 135328. https://doi.org/10.1016/j.scitotenv.2019.135328.

Liu Q, Tang X, Wang Y, Yang Y, Zhang W, Zhao Y, Zhang X (2019) ROS changes are responsible for tributyl phosphate (TBP)-induced toxicity in the alga Phaeodactylum tricornutum. Aquat. Toxicol. 208, 168–178. <https://doi.org/> 10.1016/j.aquatox.2019.01.012.

Luo Q, Gu LY, Wu ZP, Shan Y, Wang H, Sun LN (2020) Distribution, source apportionment and ecological risks of organophosphate esters in surface sediments from the Liao River, Northeast China. Chemosphere, 250: 126297

Ma Y, Xie Z, Lohmann R, Mi W, Gao G (2017) Organophosphate Ester flame retardants and plasticizers in ocean sediments from the North Pacific to the Arctic Ocean. Environ. Sci. Technol. 51 (7), 3809–3815. <https://doi.org/10.1021/acs.est.7b00755>

McGee SP, Cooper EM, Stapleton HM, Volz DC (2012) Early zebrafish embryogenesis is susceptible to developmental TDCPP exposure. Environ. Health Perspect. 120 (11), 1585–1591. <https://doi.org/10.1289/ehp.1205316>.

Mi L, Xie Z, Zhao Z, Zhong M, Mi W, Ebinghaus R, Tang J (2019) Occurrence and spatial distribution of phthalate esters in sediments of the Bohai and Yellow seas. Sci. Total Environ. 653, 792-800.

Pantelaki I, Voutsa D (2019) Organophosphate flame retardants (OPFRs): A review on analytical methods and occurrence in wastewater and aquatic environment. Sci Total Environ., 649:247-263. doi: 10.1016/j.scitotenv.2018.08.286.

Peverly AA, O’Sullivan C, Liu LY, Venier M, Martinez A, Hornbuckle KC, Hites RA (2015) Chicago’s sanitary and ship canal sediment: polycyclic aromatic hydrocarbons, polychlorinated biphenyls, brominated flame retardants, and organophosphate esters. Chemosphere 134, 380–386.

Pubchem (2022) ˂[*https://pubchem.ncbi.nlm.nih.gov*](https://pubchem.ncbi.nlm.nih.gov)˃

Sutton R, Chen D, Sun J, Greig DJ, Wu Y (2019) Characterization of brominated, chlorinated, and phosphate flame retardants in San Francisco Bay, an urban estuary. Sci. Total Environ. 652, 212-223.

Tan XX, Luo XJ, Zheng XB, Li ZR, Sun RX, Mai BX (2016) Distribution of organophosphorus flame retardants in sediments from the Pearl River Delta in South China. Sci. Total Environ. 544, 77-84.

Verbruggen EMJ, Rila JP, Traas TP, Posthuma-Doodeman CJAM, Posthumus R, (2005) Environmental risk limits for several phosphate esters,with possible application as flame retardant. <http://refhub.elsevier.com/S0160-4120(13)00129-3/rf0285>, Accessed date: 10 May 2019.

Xing LQ, Zhang Q, Sun X, Zhu HX, Zhang SH, Xu HZ (2018) Occurrence, distribution and risk assessment of organophosphate esters in surface water and sediment from a shallow freshwater Lake, China. Sci. Total Environ. 636, 632–640.

Yadav IC, Devi NL, Li J, Zhang G, Covaci A. (2018) Concentration and spatial distribution of organophosphate esters in the soil-sediment profile of Kathmandu Valley, Nepal: Implication for risk assessment. Sci. Total Environ. 613–614, 502–512

Zhang R, Yu K, Li A, Zeng W, Lin T, Wang Y (2020) Occurrence, phase distribution, and bioaccumulation of organophosphate esters (OPEs) in mariculture farms of the Beibu Gulf, China: A health risk assessment through seafood consumption. Environ. Pollut. 263, 114426.

Zhang L, Lu L, Zhu W, Yang B, Lu D, Dan SF, Zhang S (2021) Organophosphorus flame retardants (OPFRs) in the seawater and sediments of the Qinzhou Bay, Northern Beibu Gulf: occurrence, distribution, and ecological risks. Mar. Pollut. Bull. 168, 112368. <https://doi.org/10.1016/j.marpolbul.2021.112368>
